# Structural analysis of PSI-ACPI and PSII-ACPII supercomplexes from a cryptophyte alga *Rhodomonas* sp. NIES-2332

**DOI:** 10.3389/fpls.2025.1716939

**Published:** 2025-11-27

**Authors:** Wenyue Zhang, Nozomi Yonehara, Mizuki Ishii, Haowei Jiang, Romain La Rocca, Pi-Cheng Tsai, Hongjie Li, Koji Kato, Fusamichi Akita, Jian-Ren Shen

**Affiliations:** 1 Advanced Research Field, Research Institute for Interdisciplinary Science, and Graduate School of Environmental, Life, Natural Science and Technology, Okayama University, Okayama, Japan; 2 Center for Transformative Science and School of Life Science and Technology, ShanghaiTech University, Shanghai, China

**Keywords:** cryptophytes, *Rhodomonas*, photosystem I, photosystem II, light-harvesting complex, photosynthesis

## Abstract

Light energy is converted to chemical energy by two photosystems (PSI and PSII) in complex with their light-harvesting complex proteins (LHCI and LHCII) in photosynthesis. *Rhodomonas* is a member of cryptophyte alga whose LHCs contain unique chlorophyll *a/c* proteins (ACPs) and phycobiliproteins. We purified PSI-ACPI and PSII-ACPII supercomplexes from a cryptophyte *Rhodomonas* sp. NIES-2332 and analyzed their structures at high resolutions of 2.08 Å and 2.17 Å, respectively, using cryo-electron microscopy. These structures are largely similar to those reported previously from two other species of cryptophytes, but exhibited some differences in both the pigment locations and subunit structures. A part of the antenna subunits of both photosystems is shifted compared with the previously reported structures from other species of cryptophytes, suggesting some differences in the energy transfer rates from the antenna to the PSI and PSII cores. Newly identified lipids are found to occupy the interfaces between the antennae and cores, which may be important for assembly and stabilization of the supercomplexes. Water molecules surrounding three iron-sulfur clusters of the PSI core are found in our high-resolution structure, some of which are conserved from cyanobacteria to higher plants but some are different. In addition, our structure of PSII-ACPII lacks the subunits of oxygen-evolving complex as well as the Mn_4_CaO_5_ cluster, suggesting that the cells are in the S-growth phase, yet the PSI-ACPI structure showed the binding of PsaQ, suggesting that it is in an L-phase. These results suggest that the S-phase and L-phase can co-exist in the cryptophytic cells. The high-resolution structures of both PSI-ACPIs and PSII-ACPIIs solved in this study provide a more solid structural basis for elucidating the energy transfer and quenching mechanisms in this group of the organisms.

## 1 Introduction

Photosynthesis converts light energy into chemical energy by two photosystems, photosystem I (PSI) and photosystem II (PSII) (Nelson and Junge, 2015). Among them, PSII splits water and generates electrons, protons and molecular oxygen, whereas PSI transfers the electrons generated by PSII to nicotinamide adenine dinucleotide phosphate, producing reducing power to be utilized for the subsequent CO_2_ fixation. Both PSII and PSI are composed of a reaction center core associated with outside antenna proteins which function to harvest light energy and transfer them to the reaction center. While the PSI and PSII core proteins are largely conserved from prokaryotic cyanobacteria to higher plants, the antenna proteins and the pigments they bind differ greatly depending on the species of the organisms as well as the light environment they lives, which give rise to the huge diversity of photosynthetic organisms. In cyanobacteria and primitive eukaryotic red algae, the antenna proteins are hydrophilic phycobilisomes (red algae also contain transmembrane light-harvesting complex I (LHCI) proteins), which are associated at the stromal side of the thylakoid membrane and transfer the light energy they harvested to the cores of PSI and PSII (Adir et al., 2020; You et al., 2023). On the other hand, in the majority of eukaryotic organisms, the antenna proteins are trans-membrane light-harvesting complex proteins I and II (LHCI and LHCII, Pan et al., 2020; Shen, 2022; Wang and Shen, 2021; Iwai et al., 2024), which belong to the *lhc* supergene family and harvest, transfer the light energy to the PSI and PSII cores as well as dissipate excess energy under strong light conditions.

Owing to the rapid development of single particle structural analysis technique by cryo-electron microscopy (cryo-EM), the structures of both PSI-LHCI and PSII-LHCII supercomplexes have been elucidated from a variety of organisms such as diatoms (Nagao et al., 2019; Wang et al., 2019; Nagao et al., 2020; Xu et al., 2020), green algae (Qin et al., 2019; Shen et al., 2019; Sheng et al., 2019; Su et al., 2019; Suga et al., 2019; Huang et al., 2021; Tsai et al., 2025), and higher plants (Wei et al., 2016; Su et al., 2017; Yan et al., 2021; Sheng et al., 2021). These structural analyses reveal the organization and arrangement of protein subunits and pigments in the PSI-LHCI and PSII-LHCII supercomplexes, and provide structural bases for elucidating the excitation energy transfer (EET) or energy dissipation pathways from LHCs to the reaction center cores or vice versa. They also provided clues to the evolutionary changes occurred in both the proteins and pigments of the LHC systems from primitive algae to higher plants. For example, the PSI-LHCI from a diatom contains up to 24 antenna subunits arranged in three layers surrounding the PSI core (Xu et al., 2020), which constitutes one of the largest PSI-LHCI supercomplexes studied so far. The antennas of diatoms bind chlorophylls (Chls) *a, c*, and fucoxanthins, and are thus termed FCPs (fucoxanthin Chl *a/c* binding proteins) (Wang et al., 2019). These pigments form a huge, complicated network within PSI-FCPI of diatoms, which not only efficiently harvest the light energy and transfer them to the PSI core, but also function to dissipate excess energy under strong light illumination conditions.

Cryptophytes are unicellular algae originated from a primitive red alga via secondary endosymbiosis, and possess unique pigments (Raven and Giordano, 2014; Cunningham et al., 2019). The antenna system of red algae consists of LHCI and extramembrane phycobiliproteins (Pi et al., 2018; You et al., 2023; Kato et al., 2024). Different from the red algal antennae, cryptophyte LHCs contain a unique carotenoid alloxanthin (Alx), as well as Chl *a*/*c*_2_, which are therefore termed ACP (alloxanthin and Chl *a*/*c* binding protein) (Zhao et al., 2023; Mao et al., 2024) or CAC (Chl *a*/*c* binding protein) (Kuthanová Trsková et al., 2019; Zhang et al., 2024a) instead of LHC.

Recently, the structure of a cryptophyte PSI-ACPIs supercomplex from *Chroomonas placoidea* (*C. placoidea, Cp*) was analyzed by cryo-EM at a 2.7 Å resolution, which showed the association of either 11 or 14 ACPI subunits around the PSI core, as well as an unknown subunit named Unk1 (PDB code: 7Y7B) (Zhao et al., 2023). This subunit was identified as PsaQ in the structure of another cryptophyte *Rhodomonas salina* (*Rs*) analyzed at a similar resolution (PDB code: 8WM6) (Zhang et al., 2024a). The growth of cryptophytes undergoes transition from logarithmic phase in the early time to stationary phase at a later time (named L-phase and S-phase, respectively), and PsaQ was found to exist in the L-phase only and thus may assist the association of phycobiliproteins to photosystems and facilitate energy transfer between them (Zhang et al., 2024a). The same group also published the structure of PSII-ACPIIs from *R. salina* purified from cells at nitrogen-limited S-phase at 2.57-Å resolution, which showed a homo-dimeric organization of PSII-ACPIIs with each PSII monomeric core associated with 6 ACPII subunits (PDB code: 8XLP) (Si et al., 2024). The extrinsic subunits of the oxygen-evolving complex were lacking together with the catalytic center for water oxidation, the Mn_4_CaO_5_ cluster, which was explained to be due to the nitrogen depletion condition in the S-phase. The structure of PSII-ACPIIs was further solved from *C. placoidea* cells grown under L-phase, which showed a similar association of 6 ACPII subunits with each PSII monomer core, but the structure retained three extrinsic subunits of PsbU, PsbV, PsbO, and the Mn_4_CaO_5_ cluster, suggesting a complete and active PSII structure in the L-phase (PDB code: 8WB4 and 8XR6) (Mao et al., 2024; Zhang et al., 2024b). In these PSII-ACPII structures, a subunit called CCPII-S or Psb-γ was found, which exists between the ACPII antenna and PSII core, and therefore may mediate the binding and interaction of ACPII to the PSII core. Because CCPII-S also binds pigments, it may play possible roles in energy transfer from ACPII to the core.

In order to analyze the PSI-ACPIs and PSII-ACPIIs structures of cryptophytes in more detail, we purified these supercomplexes from another species of cryptophyte *Rhodomonas* sp. NIES-2332 (*R.* sp. NIES-2332) closely related to *R. salina*, and solved the structures of PSI-ACPIs and PSII-ACPIIs at higher resolutions of 2.08 Å and 2.17 Å, respectively, by cryo-EM. Our high-resolution structures revealed more structural details, including some protein regions that are not solved previously or different from other species, and pigments, lipids, detergent molecules that were not visible in the previous structures. Interestingly, PsaQ was found in the PSI-ACPIs structure, suggesting that the cells are in the L-phase, yet the PSII-ACPIIs structure showed the absence of the extrinsic proteins and the Mn_4_CaO_5_ cluster, suggesting that the cells are in the S-phase. These results suggest a mixture of L-phase PSI-ACPIs and S-phase PSII-ACPIIs in the same cells, which provide more detailed structural information on PSI-LHCIs and PSII-LHCIIs of cryptophytes, as well as a more solid structural basis for elucidating the energy transfer and quenching mechanisms in this group of organisms.

## 2 Materials and methods

### 2.1 Purification of the PSI-ACPI and PSII-ACPII supercomplexes

*Rhodomonas* sp. NIES-2332 cells were cultured in an F/2 medium (Guillard and Ryther, 1962) bubbled with air containing 3% CO_2_ under constant LED light at 20°C. Cells were harvested at around 10 days, when their OD_730_ reached to 1.0. The cells harvested were disrupted by French press at a pressure of 60 MPa for 6 cycles. Thylakoid membranes were recovered by centrifugation, and solubilized by 0.9% n-dodecyl-α-D-maltoside (α-DDM) (Anatrace) in a buffer MES-1 (25 mM MES–NaOH, pH 6.5, 1.0 M betaine, 10 mM NaCl, 5 mM CaCl_2_) for 30 min. The solubilized membranes were loaded onto a discontinuous sucrose density gradient with a sucrose concentration from 10% to 30%, with an interval of 2% in buffer MES-1 containing 0.03% α-DDM, and centrifuged at 230,000 *x* g for 20 hrs. The bands of PSI-ACPIs and PSII-ACPIIs (**Supplementary Figure S1**) were collected and concentrated by an Amicon Ultra 100 kDa filter (Millipore) in buffer MES-2 (25 mM MES–NaOH, pH 6.5, 50 mM NaCl, 5 mM CaCl_2_, 0.03% α-DDM).

### 2.2 Characterization of PSI-ACPI and PSII-ACPII supercomplexes

Absorption spectra were measured at room temperature by a UV-vis spectrophotometer (UV-2450, Shimadzu). Sodium dodecyl sulfate (SDS)-polyacrylamide gel electrophoresis (PAGE) was performed with a gel containing 16% polyacrylamide and 7.5 M urea (Ikeuchi and Inoue, 1988). The sample was treated with a buffer containing 2% (w/v) lithium lauryl sulfate, 60 mM dithiothreitol and 60 mM Tris-HCl (pH 8.5) at 60°C for 20 min before loaded onto the gel, and the resulted gel was stained with Coomassie brilliant blue (CBB) R-250.

### 2.3 Sequence analysis of the PSI-ACPI and PSII-ACPII components

Cells were collected, suspended in RNAlaterTM (Thermofisher Scientific), and immediately frozen in liquid nitrogen for storage at -80 °C. Total RNA extraction, cDNA library construction, sequencing and bioinformatics analysis were conducted by Bioengineering Lab Co., Ltd (La Rocca et al., 2025). Sequences of the PSI and PSII core and antennae subunits were identified by blast analysis against the National Center for Biotechnology Information database.

### 2.4 Cryo-EM data collection

An aliquot of 4 μL of the sample containing both PSI-ACPI and PSII-ACPII supercomplexes at a Chl *a* concentration of 1.0 mg mL^-1^ was applied to a glow-discharged holey carbon grid (Quantifoil R1.2/1.3 Cu 300 mesh). The grid was blotted for 5 s with the force level of 10 at 6 °C and 100% humidity and plunged into liquid ethane which was already cooled by liquid nitrogen in an FEI Vitrobot Mark IV chamber. The grid was loaded onto a 300 kV Titan Krios G4 microscope equipped with a Falcon 4i camera for data collection. A total of 29,410 movie stacks were recorded by EPU (Thermo Fisher) at a total dose of 50 e^-^Å^-2^ for each stack movie with a defocus range of -0.6 ~ -1.8 μm, with the magnification of ×165,000 corresponding to a pixel size of 0.727 Å.

### 2.5 Data processing

All movie stacks were corrected by Patch Motion Correction and patch contrast transfer function (CTF) estimation (Rohou and Grigorieff, 2015; Zheng et al., 2017). Data processing was performed by cryoSPARC 4.6.2 (Punjani et al., 2017). PSI-ACPI particles were picked from 20,115 micrographs, and PSII-ACPII particles were picked from 29,410 micrographs. Particles resulted from automatic picking were subjected to 2D and 3D classifications. After 3D non-uniform refinement, local CTF refinement and reference-based motion correction, final maps were obtained, which showed resolutions of 2.08 Å and 2.17 Å for PSI-ACPI and PSII-ACPII, respectively. To improve the local resolutions of the density maps of some antennae parts, local refinement was performed. Local refinement targeting the ACPI-d/e/f/g, ACPI-i/j/k, and ACPI-l/m/n, resulted in local maps with resolutions of 2.67 Å, 2.66 Å and 2.64 Å, respectively. Local refinement targeting the ACPII part resulted in a local map of 3.25 Å. These local maps are used to refine the models of the corresponding local structures. The resolution was estimated based on the gold-standard Fourier shell correlation of 0.143 (Grigorieff and Harrison, 2011).

### 2.6 Model building and refinement

For model building, the PSI-ACPI and PSII-ACPII structures of *R. salina* (PDB:8WM6 from Zhang et al., 2024a and 8XLP from Si et al., 2024) were manually placed and rigid-body-fitted into the 2.08 Å and 2.17 Å density maps with ChimeraX (Pettersen et al., 2004). Subsequently, amino acid residues were mutated to their counterparts in *Rhodomonas* sp. NIES-2332, with the sequences obtained from the cDNA sequencing results. The sequences of PsaA, PsaB, PsaD, PsaF, PsaL, PsaR, PsaO, PsaQ, ACPI-s and all ACPIs subunits were found in the sequencing results, whereas PsaC, PsaE, PsaI, PsaJ, PsaM, PsaK were missing in the sequencing results and the sequences of these missing subunits were taken from *R. salina* (8WM6) (Zhang et al., 2024a) in PSI model building. On the other hand, PsbA, PsbB, PsbC, PsbD, PsbM, PsbW, Psb-γ, ACPII-1/2/3/4/5/6 sequences were found in the sequencing results for PSII-ACPII, whereas the sequences of the remaining PSII subunits were not found in the sequencing results, so these sequences are taken from *R. salina* (8XLP) (Si et al., 2024) in the PSII-ACPII model building. After model building, automatic real space refinements were carried out with Phenix (Adams et al., 2010) and manual correction was carried out with COOT (Emsley et al., 2010) iteratively. The geometries of the structures were assessed using Phenix and MolProbity (Adams et al., 2010; Chen et al., 2010), and the detailed information was listed in **Supplementary Table S1**. The correspondences of the names of ACPI and ACPII subunits used in the present paper with those in the PDB file as well as the other two species of cryptophytes are listed in **Supplementary Tables S2** and **S3**. The structure figures were prepared with ChimeraX 1.8 (Pettersen et al., 2004).

### 2.7 Förster energy transfer rate calculation

The Förster energy transfer (FRET) rate *K_DA_* is defined as *K_DA_=C_DA_k^2^/n^4^*Rê_DA_*^6^* according to the FRET theory (Gradinaru et al., 1998), where *C* is a factor calculated from the overlap integral between the two Chls, *K* is the dipole orientation factor, *n* is the refractive index and *R* is the distance between two central magnesium atoms of Chls. A *C* value of 32.26 was applied for Chl *a* → Chl *a* energy transfer, and an *n* value of 1.55 was taken from Gradinaru et al. (1998). *k*^2^ is the dipole orientation factor, defined as *k^2^*=[û_D_·û_A_−3·(û_D_·Rê_DA_)·(û_A_·Rê_DA_)]^2^, where û_D_ and û_A_ are the transition dipole unit vectors (taken as the vector between the N_B_ and N_D_ atoms of the specific Chl *a* molecule), and Rê_DA_ is the distance between the two pigments (taken as the magnesium to magnesium distance between each pair of Chls). The FRET rates were computationally calculated using Kim’s algorithm available at https://doi.org/10.5281/zenodo.3250649 (Kim, 2019), and only Chl *a* → Chl *a* energy transfer is considered here, because the *C* value involving Chl *c* is not known at present.

## 3 Results

### 3.1 Overall structures

#### 3.1.1 PSI-ACPIs

**Figure 1:**
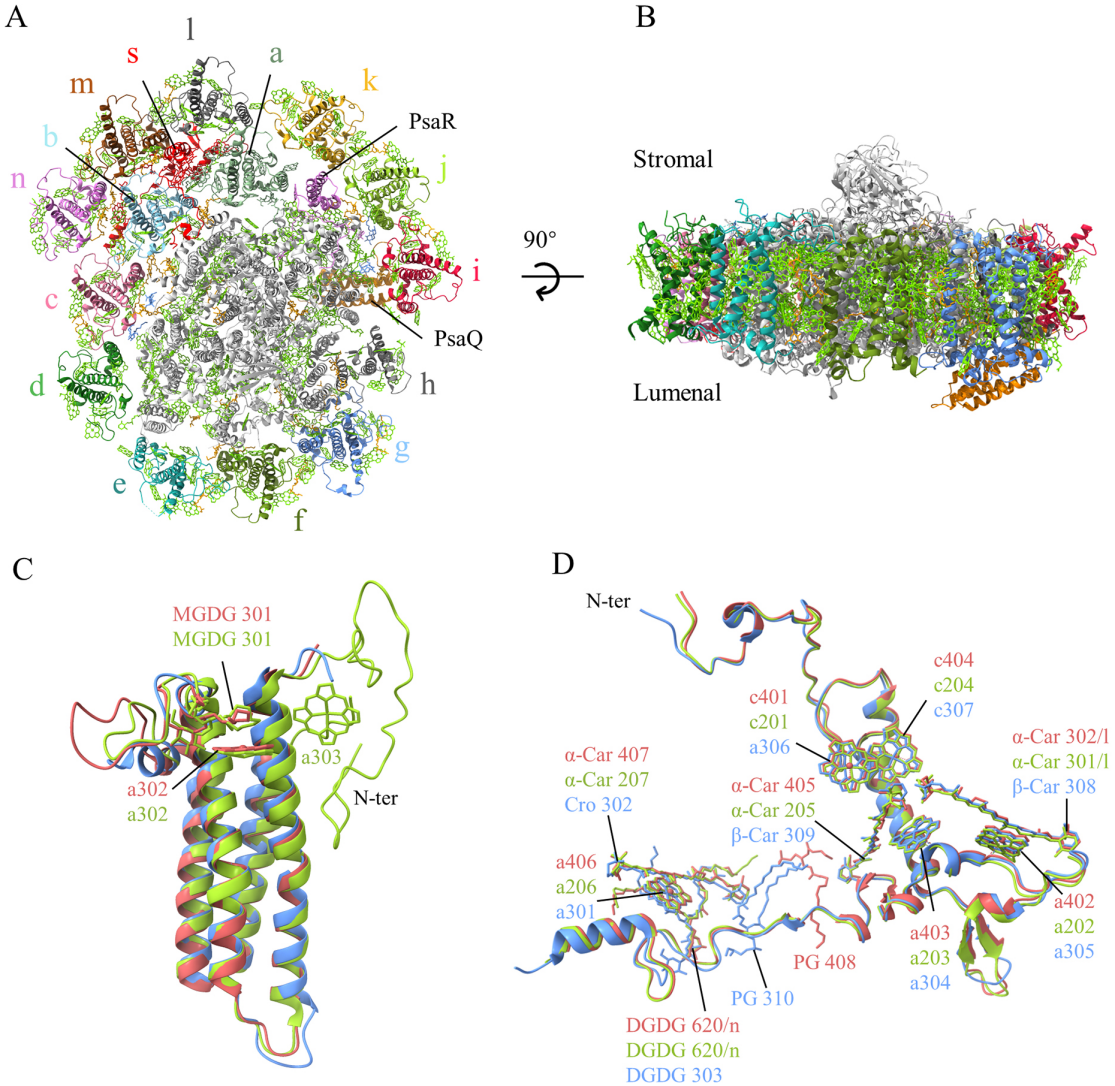
**(A)** Overall structure of the PSI-ACPI supercomplex viewed from the stromal side. **(B)** Side view of the supercomplex. Lawn green: chlorophylls and carotenoids, orange: lipids. **(C, D)** Superposition of the PsaQ **(C)** and ACPI-s **(D)** subunits from the three structures of *Rhodmonas* sp. NIES-2332 (Indian red), *Rhodomonas salina* (yellow green), and *Chroomonas placoidea* (cornflower blue).

PSI-ACPIs were purified from *Rhodomonas* sp. NIES-2332 (**Supplementary Figure S1**; Materials and Methods section), and its structure was solved by cryo-EM, which showed that it contained two supercomplexes, one with 14 ACPIs and another one with 11 ACPIs (**Figure 1**; **Supplementary Figures S2**-**S4**; **Supplementary Table S1**). The structures of PSI-14 ACPIs and PSI-11 ACPIs are solved at resolutions of 2.08 and 2.14 Å, respectively (**Supplementary Table S1**). Like the previously reported structures (Zhao et al., 2023; Zhang et al., 2024a), the PSI core contained 14 subunits (PsaA-F, PsaI-M, PsaO, PsaQ and PsaR) (**Figure 1**). While PsaR is a subunit unique to cryptophyte, PsaQ was assigned as Unk1 and represented by poly-alanines in the structure of *C. placoidea* (Zhao et al., 2023), and its sequences were identified only in the L-phase structure of *R. salina* but lost in the S-phase structure of *R. salina* (Zhang et al., 2024a). This suggests that the structure we solved is also in the L-phase.

PsaQ is a hydrophilic subunit located at the lumenal side, with a four helices-bundle structure similar to that of PsbQ found in PSII (**Figure 1B**). The structure of PsaQ in *R.* sp. NIES-2332 is similar to that in *C. placoidea* and does not have an extra N-terminal region, whereas it has an extra N-terminal region in *R. salina* (Zhang et al., 2024a) (**Figure 1C**). Interestingly, it binds a Chl *a* at the membrane surface (*a*302/Q), which is close to PsaB but without an amino acid residue as its ligand. This Chl *a* is present in the *R. salina* structure also (Zhang et al., 2024a), but absent in the structure of *C. placoidea* (Zhao et al., 2023) (**Figure 1C**). In addition, a monogalactosyldiacylglycerol molecule (MGDG301) was found in the PsaQ structure of both *R.* sp. NIES-2332 and *R. salina*, but was absent in *C. placoidea*, and another Chl *a* (*a*303/Q) was found near Chl *a*302 in the PsaQ structure of *R. salina* but was absent in both *R.* sp. NIES-2332 and C. *placoidea* structures (**Figure 1C**).

In the PSI-14 ACPIs supercomplex, 14 ACPIs are named ACP-a to ACP-n, and another subunit ACPI-s is also found (**Figure 1A**), whereas the PSI-11 ACPIs supercomplex lacks three ACPIs, namely, ACPI-e/f/g (**Supplementary Figure S4**). As the PSI-11 ACPI structure has also been found in the previous study (Zhang et al., 2024a), we consider that this represents a form of PSI-ACPIs present *in vivo*, but we cannot exclude that a part of ACPIs have been released from PSI due to detergent solubilization during isolation, leading to the observation of the PSI-11 ACPI supercomplex.

The 14 ACPI subunits form two layers surrounding the PSI core, with the inner layer composing of 11 ACPI subunits (ACP a-k) and the out layer composing of only 3 subunits (ACP l-n). Like the previously reported structures (Zhao et al., 2023; Zhang et al., 2024a), ACPI-s is a single trans-membrane helix subunit and occupies the space between the inner layer (ACP a-b) and out layer (ACP l-m), with its long N-terminal region spanning across the inner layer and approaching to the core at the stromal side, and its C-terminal region spanning across both the inner and outside layer of the antenna (**Figure 1D**). The pigments bound to ACPI-s are the same among the three species, except that two α-carotenes assigned in *R*. sp. NIES-2332 and *R*. *salina* were changed to β-carotene (β-car 308, 309), a Chl *c* (Chl c401 in *R.* sp. NIES-2332 and c201 in *R. salina*) was changed to Chl *a*, and an α-carotene (α-Car 407 in *R*. sp. NIES-2332 and α-Car 207 in *R*. *salina*) was changed to a Cro (Cro302), in *C. placoidea* (**Figure 1D**). In addition, one phosphatidyl glycerol (PG) molecule found in *C. placoidea* shifted its position in *R.* sp. NIES-2332, but this PG is not found in *R. salina* (**Figure 1D**). It appears that ACPI-s is a subunit connecting the two antenna layers and also for the binding of the antenna subunits to the PSI core.

In addition to the protein subunits, we found 255 Chls *a*, 20 Chls *c*, 58 Alx, 29 α-carotene (α-Car), 12 crocoxanthin (Cro), 6 detergent molecules, as well as 44 phosphatidylglycerol (PG), 17 MGDG, 3 digalactosyldiacylglycerol (DGDG), 1 sulfoquinovosyldiacylglycerol (SQDG), and 422 water molecules in the *R.* sp. NIES-2332 PSI-ACPI structure.

**Figure 2:**
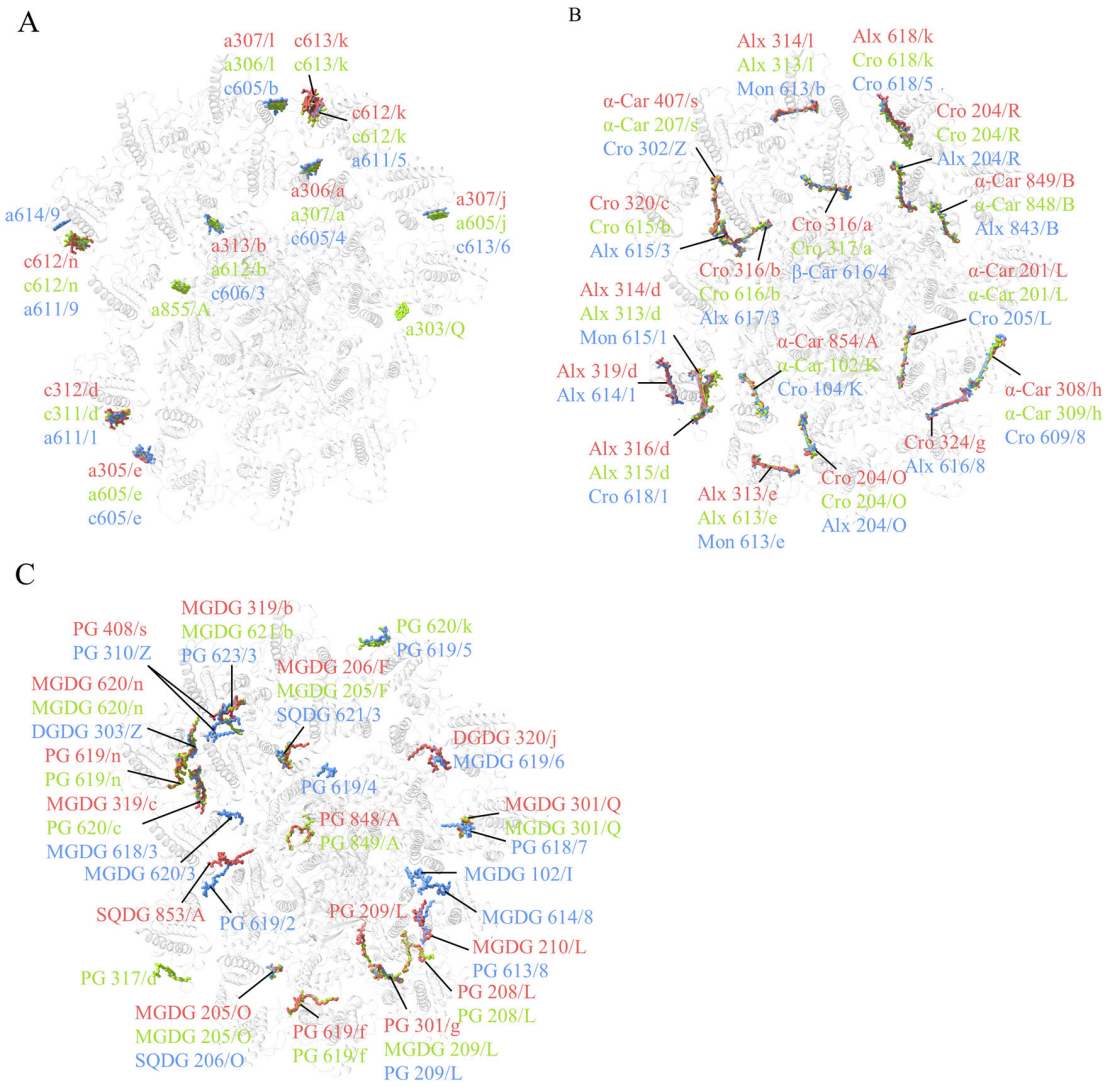
Comparison of chlorophylls **(A)**, carotenoids **(B)** and lipids **(C)** in the three PSI-ACPIs structures of cryptophytes, *Rhodmonas* sp. NIES-2332 (Indian red), *Rhodomonas salina* (yellow green), *Chroomonas placoidea* (coenflower blue). Only chlorophylls, carotenoids and lipids that are assigned differently in the three structures are shown, whereas those pigments and lipids that are assigned the same in the three structures are omitted.

Compared with the structure of *R. salina* (8WM6) (Zhang et al., 2024a), Chl *a*855/PsaA is missing in both *R.* sp. NIES-2332 and *C. placoidea* structures in addition to *a*303/PsaQ (**Figure 2A**; **Supplementary Table S4**). In the ACPI antennae, 10 Chl binding-sites were found to be different among the three structures reported. Among them, *C. placoidea* lacks one Chl *c* site (c613/k) that existed in *R.* sp. NIES-2332 and *R. salina*, whereas some sites (Chl a306/a, a313/b, a305/e, a307/l, a307/j) are assigned as Chl *a* in *R.* sp. NIES-2332 but they are Chl *c* in *C. placoidea*, and other sites (Chl c312/d, c612/k, c612/n) are assigned as Chl *c* in *R.* sp. NIES-2332 but they are Chl *a* in *C. placoidea* (**Figure 2A**; **Supplementary Table S4**). Finally, Chl a614/9 was found in *C. placoidea* only, but it was absent in both *R.* sp. NIES-2332 and *R. salina* (**Figure 2A**; **Supplementary Table S4**). These Chls are located largely in the peripheral region of the antenna subunits which appear to be more variable than the PSI core pigments, and some of them may be miss-assigned in some structures due to lower resolutions.

Most carotenoid-binding sites are present in the three organisms compared, except Alx 319/d and Cro 324/g which are present in both *R*. sp. NIES-2332 and *C. placoidea* but absent in *R. salina* (**Figure 2B**; **Supplementary Table S4**). However, at the same carotenoid-binding sites, the types of carotenoids appear to be different among the three organisms. For example, 4 α-carotenes (α-Car 854/A, 308/h, 201/L, 407/s) assigned in *R.* sp. NIES-2332 as well as in *R. salina* are assigned as crocoxanthins (Cro 104/K, 609/8, 205/L, 320/Z), in *C. placoidea* (**Figure 2B**; **Supplementary Table S4**). All Alxs (Alx 314/d, 313/e, 314/l) assigned in *R*. sp. NIES-2332 and *R. salina* became monadoxanthins (Mon 615/1, 613/e, 613/b) in *C. placoidea*, and 5 crocoxanthins (Cro 204/O, 316/b, 320/c, 204/R, 324/g) assigned in *R.* sp. NIES-1332 (among which, 4 are also present in *R. salina* and only Cro 324/g is absent in *R. salina*) are assigned as Alxs (Alx 204/O, 615/3, 617/3, 204/R, 616/8) in *C. placoidea*. (**Figure 2B**; **Supplementary Table S4**). These differences may be due to species differences, but some of them may be due to miss-assignment at lower resolutions.

Two lipids (SQDG 853/A and PG 209/L) are newly found in the structure of *R*. sp. NIES-2332, among which, a PG molecule (PG 619/2) is found in a position close to SQDG 853/A in *C. placoidea* (**Figure 2C**). Three MGDG (MGDG 102/I, 620/3, 614/8) and 1 PG (619/4) molecules are found in *C. placoidea* only, and they are absent in the structures of *R.* sp. NIES-2332 and *R. salina*. One PG molecule (317/d) is found in *R. salina* only, and some other lipids are absent in one of the three species but present in other two species (**Figure 2C**).

#### 3.1.2 PSII-ACPIIs

**Figure 3:**
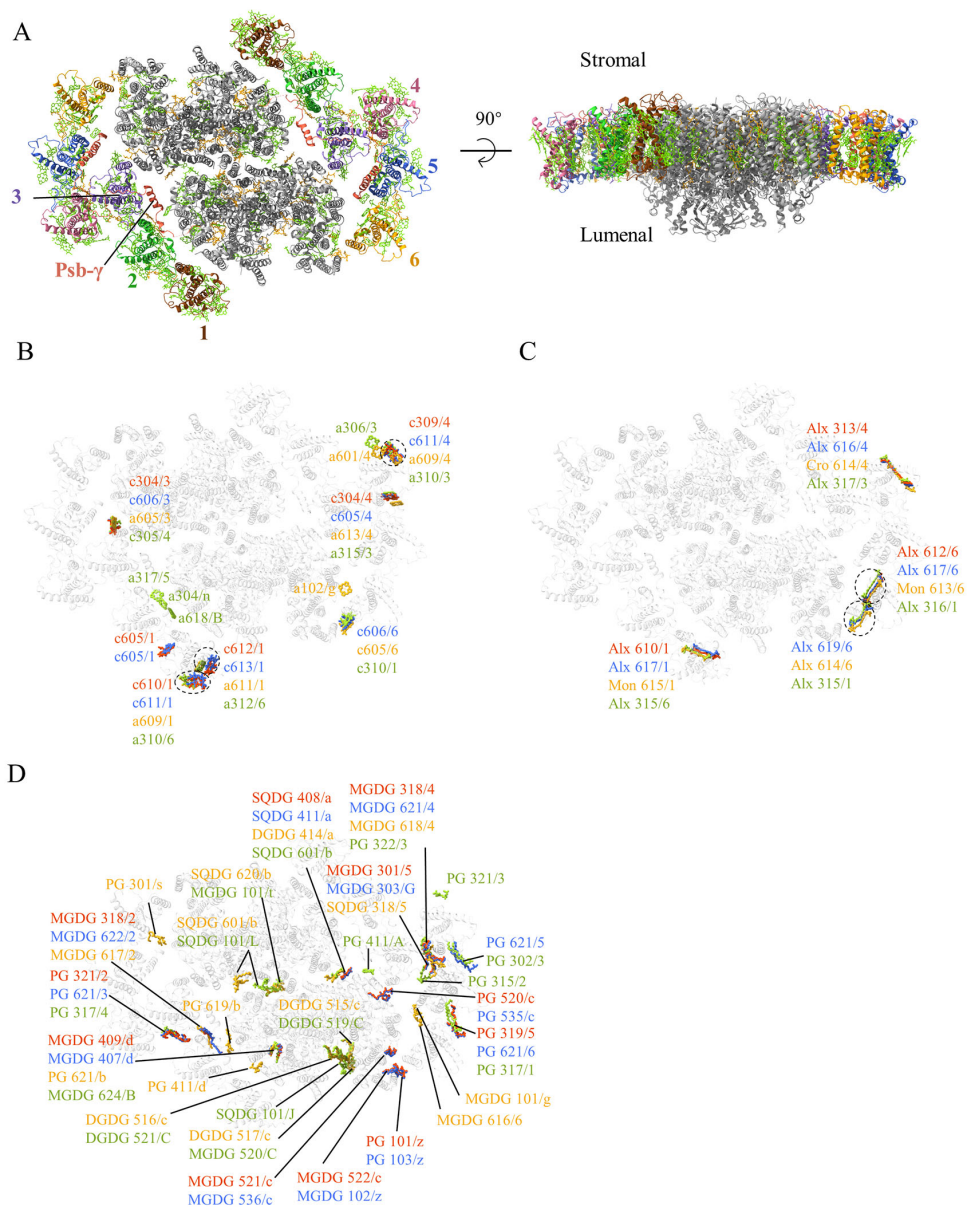
**(A)** Overall structure of the PSII-ACPII supercomplex with a top view from the stromal side (left side) and a side view (right side). **(B–D)** Comparison of chlorophylls **(B)** carotenoids **(C)** and lipids **(D)** in the three structures of cryptophytes, *Rhodmonas* sp. NIES-2332 (Indian red), *Rhodomonas salina* (royal blue: 8XLP from Si et al., 2024), *Chroomonas placoidea* (goldenrod: 8WB4 from Mao et al., 2024 and yellow green: 8XR6 from Zhang et al., 2024b). Only chlorophylls, carotenoids and lipids assigned differently in the three structures are shown, whereas those pigments and lipids that are assigned the same in the three structures are omitted.

PSII-ACPIIs were found in the same fraction of PSI-ACPs (**Supplementary Figure S1**), so particles corresponding to PSII-ACPIIs were picked and its structure was solved at a resolution of 2.17 Å (**Supplementary Figure S5**; **Supplementary Table S1**). As in the previous structure (Mao et al., 2024; Si et al., 2024; Zhang et al., 2024b), 6 antennae subunits (ACPII-1 to 6), together with a linker protein Psb-γ (CCPI-S in Mao et al., 2024 or CAL-II in Si et al., 2024), are found in the PSII-ACPIIs structure (**Figure 3A**). Psb-γ is located in a space between two antennae subunits APCII-2 and 3, and connects these antenna subunits to the PSII core subunit PsbB (**Figure 3A**). Compared with the structures of *C. placoidea* (8WB4, 8XR6) (Mao et al., 2024; Zhang et al., 2024b), the present structure lacks several PSII core subunits, which include PsbJ, PsbO, PsbU, PsbQ, PsbV and the Mn_4_CaO_5_ cluster functioning as the catalyst for water oxidation (Umena et al., 2011), as well as Unk1/2/3 found in the *C. placoidea* structure solved at 2.47 Å resolution (8WB4) (Mao et al., 2024) (**Supplementary Figures S6A, B**). However, our present structure is similar to the structure of *R. salina* solved at S-phase under an N-limiting condition (8XLP) (Si et al., 2024) (**Supplementary Figure S6C**), which also lacks the PsbJ, PsbO, PsbU, PsbQ and PsbV subunits and the Mn_4_CaO_5_ cluster.

In addition to the protein subunits, the whole complex contains 194 Chl *a*, 22 Chl *c*, 46 Alx, 24 α-Car, 8 Cro, as well as 18 PG, 22 MGDG, 4 DGDG, 4 SQDG, and 676 water molecules.

Among Chls, 1 Chl *a* (a102/g in Unk3) is found in a structure of *C. placoidea* only (Mao et al., 2024), 3 Chls *a* (a618/B in CP47, a317/5 in ACPII-5 and a304/n in Psb-γ) are found in another structure of *C. placoidea* (Zhang et al., 2024b), and two Chls *a* (a306/3 or a601/4) are found in both structures of *C. placoidea*, but they are absent in the structures of *R.* sp. NIES-2332 and *R. salina* (**Figure 3B**; **Supplementary Table S5**). One Chl *c* site (c605/1) is found only in the two species of *R*. sp. NIES-2332 and *R. salina*, but absent in the structures of *C. placoidea*, and another Chl *c* site (c606/6 in *R. salina* (Si et al., 2024), c605/6 in 8WB4 (Mao et al., 2024), or c310/1 in 8XR6 (Zhang et al., 2024b)) is present in the three structures of *R. salina* and *C. placoidea* but absent in the structure of *R*. sp. NIES-2332 (**Figure 3B**; **Supplementary Table S5**). In addition, 4 Chls *a* (*a*609/1, *a*611/1 in ACPII-1, *a*613/4, *a*609/4 in ACPII-4) belonging to the ACPII-1 and ACPII-4 subunits in *C. placoidea* (8WB4) (Mao et al., 2024) are replaced by Chls *c* in *R. salina* and *R*. sp. NIES-2332.

Among the carotenoids, 3 Alxs are assigned in three structures of *R.* sp. NIES-2332 (this study), *R. salina* (Si et al., 2024) and *C. placoidea* (Zhang et al., 2024b), but they are assigned as Mon in one structure of *C. placoidea* (Mao et al., 2024) (**Figure 3C** and **Supplementary Table S5**). In addition, the Alx 619/6 in *R. salina* (Si et al., 2024), Alx614/6 in *C. placoidea* (8WB4) (Mao et al., 2024), or Alx315/1 in another structure of *C. placoidea* (8XR6) (Zhang et al., 2024b), is not found in the present structure (**Figure 3C**; **Supplementary Table S5**).

PSII-ACPII structures from *R*. sp. NIES-2332 and *R. salina* showed less lipids than *C. placoidea.* For example, one structure of *C. placoidea* (8WB4) (Mao et al., 2024) showed 3 additional PG (PG 301/s, 619/b, 411/d) and 2 MGDG (MGDG 101/g, 616/6), and another structure of *C. placoidea* (8RX6) (Zhang et al., 2024b) showed 3 additional PG (PG 411/A, 321/3, 315/2) and 1 SQDG (SQDG 101/J), which are all absent in the structures of *R*. sp. NIES-2332 and *R. salina* (**Figure 3D**). Several SQDG or MGDG molecules found in both structures of *C. placoidea* are absent in *R*. sp. NIES-2332 and *R. salina* (**Figure 3D**). The lipids in *R. salina* and *R*. sp. NIES-2332 are basically identical to each other, but they contain 2 additional PG (PG 101/z, 520/c in *R.* sp. NIES-2332, or PG 103/z, 535/c in *R. salina*) and 2 MGDG (MGDG 521/c, 522/c in *R.* sp. NIES-2332, or MGDG 536/c, 102/z in *R. salina*) that are not found in *C. placoidea* (**Figure 3D**). All these differences may be due either to species differences or to miss-assignment in some of the structures due to lower resolutions.

### 3.2 Shift of subunits

#### 3.2.1 PSI-ACPIs

Among the PSI core subunits, PsaQ shifted its position toward the ACPI-i/j/k side compared with that in *R. salina* (**Figures 1A, B**; **Supplementary Figure A7**). The end of the helix neighboring the membrane side (near the ACPI-i side) shifts more than the other three helices (**Supplementary Figure S7**). This shift altered the location of PsaQ relative to the *R. salina* at the lumenal side.

**Figure 4:**
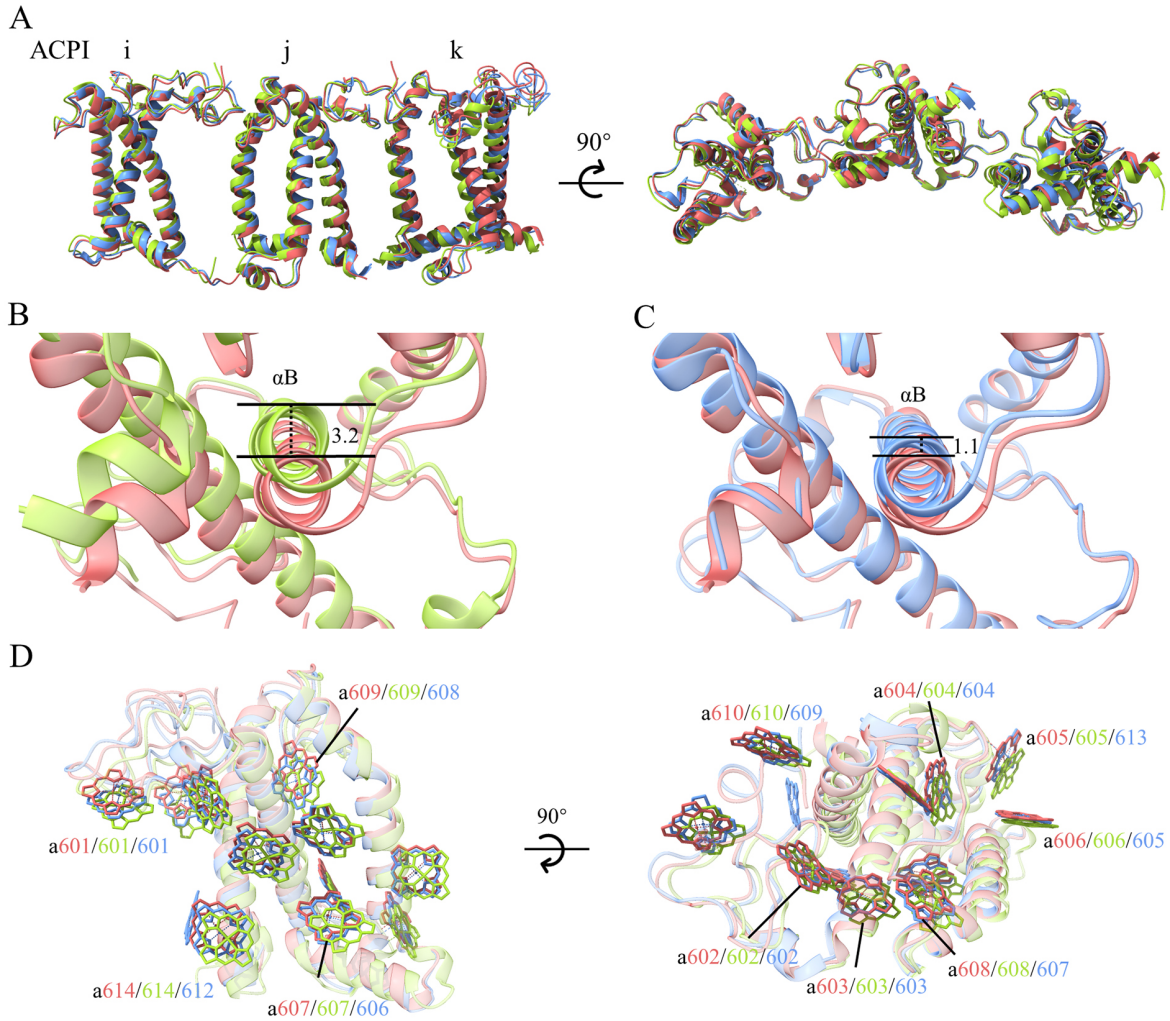
Shifts of ACPI subunits in the PSI-ACPI structures of cryptophytes. **(A)** Superposition of the ACPI-i/j/k subunits between the structures of *Rhodmonas* sp. NIES-2332 (Indian red), *Rhodomonas salina* (yellow green), *Chroomonas placoidea* (cornflower blue), with a side view shown in the left and a top view from the lumen shown in the right. **(B)** Shift of helix B of ACPI-k between *R.* sp. NIES-2332 (the present structure, Indian red) and *R. salina* (yellow green). **(C)** Shift of helix B of ACPI-k between the present structure and *C*. *placoidea* (cornflower blue). Both panels **(B, C)** are viewed from the lumenal side. **(D)** Shifts of Chls of ACPI-k from the three structures.

All ACPIs are grouped as four heterotrimers: ACPI-b/c/d, e/f/g, i/j/k and l/m/n (Zhang et al., 2024a). Among these heterotrimers, the positions of ACPI-e/f/g are almost the same as those reported previously; however, there are some shifts in the remaining subunits. Among them, ACPI-k showed the largest shift, as it is translocated outside from the PSI core, with a translocation of 3.2 Å compared with the structure of *R. salina* (Zhang et al., 2024a), and 1.1 Å compared with the structure of *C*. *placoidea* (Zhao et al., 2023) (**Figures 4A–C**). Due to these shifts, Chls bound to ACPI-k were also shifted (**Figure 4D**).

#### 3.2.2 PSII-ACPIIs

**Figure 5:**
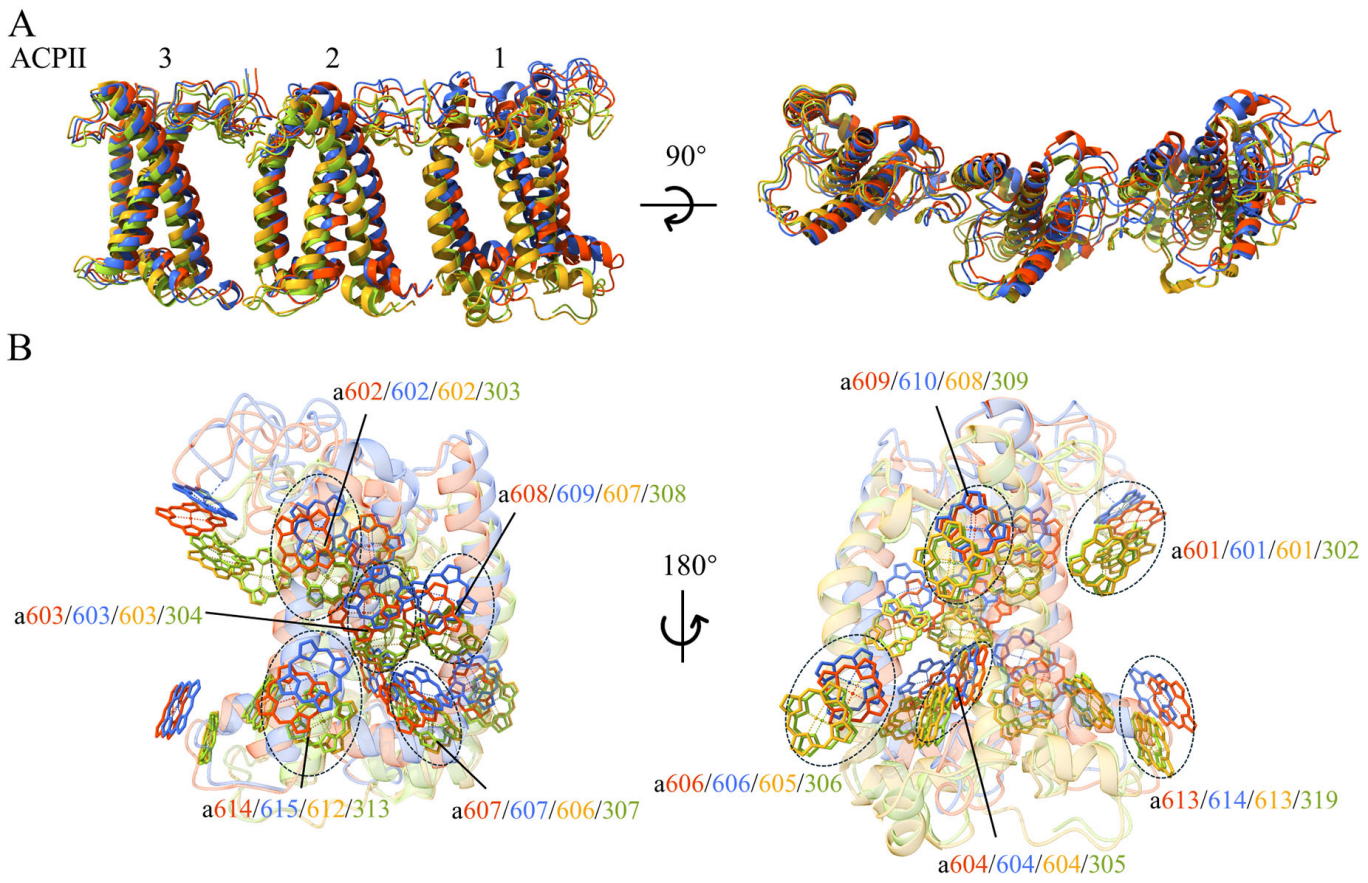
**(A)** Shifts of ACPII-1/2/3 in the different structures of cryptophytes: *Rhodmonas* sp. NIES-2332 (Indian red, this study), *Rhodomonas salina* (royal blue: 8XLP from Si et al., 2024), *Chroomonas placoidea* (goldenrod: 8WB4 from Mao et al., 2024, and yellow green: 8XR6 from Zhang et al., 2024b). **(B)** Differences in the positions of Chls assigned in the four structures of ACPII-1.

The antennae subunits of PSII also have some shifts compared with the structures reported so far. The inner layer antennae subunits ACPII-1/2/3 in *R*. sp. NIES-2332 is similar to those of *R. salina*, but shifted to become closer to the PSII core compared with those in *C. placoidea* (**Figure 5A**). ACPII-1 shifted to a larger extent than the ACPII-2/3 subunits. Due to these shifting subunits, the Chl-binding sites on these subunits are also translocated, with their translocating directions consistent with the shifted direction of the antenna subunits (**Figure 5B**). These shifts result in changes of the distances from antenna Chls to the core, which may alter the excitation energy transfer efficiencies.

### 3.3 Water molecules

#### 3.3.1 PSI-ACPIs

**Figure 6:**
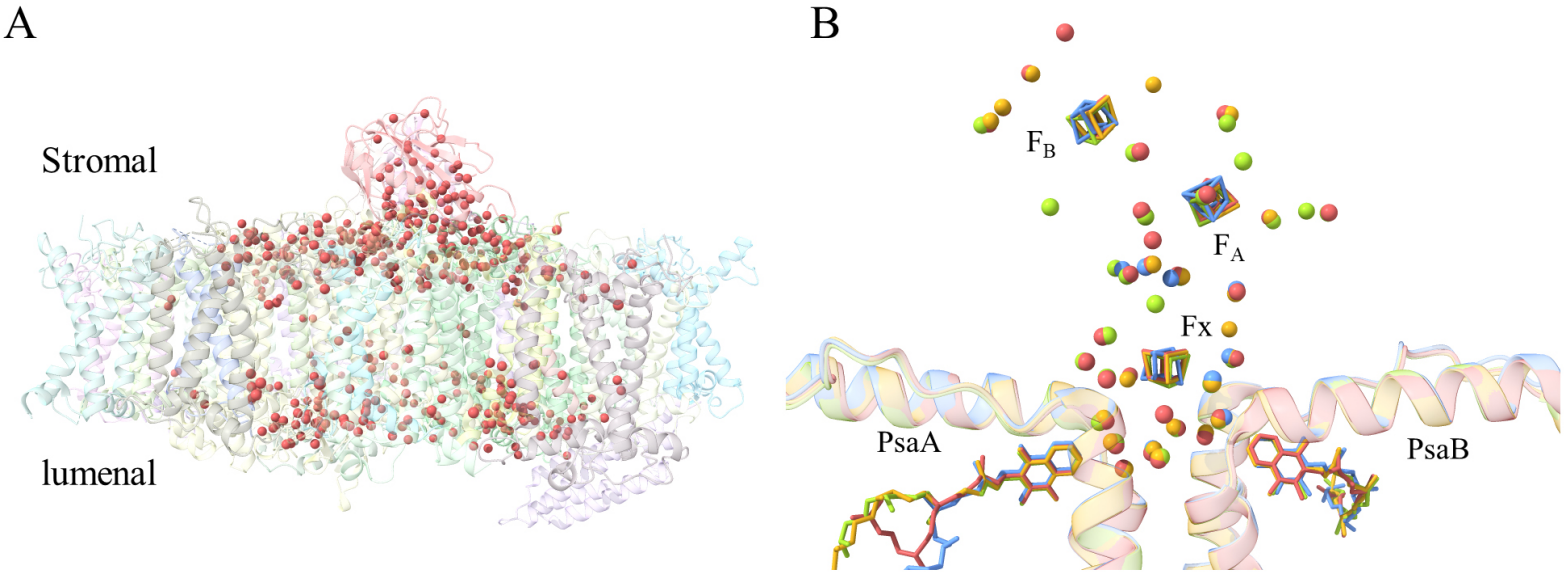
Water molecules identified in *R.* sp. NIES-2332 PSI-ACPI. **(A)** More than 400 water molecules (Indian red spheres) shown in the side view of the PSI-ACPI structure. **(B)** Close view of the conserved water molecule surrounding F_X_, F_A_ and F_B_. Water molecules found in the present structure within 8 Å of the F_X_, F_A_ and F_B_ are colored by Indian red, whereas those in the structures of cyanobacterial PSI (PDB ID: 4KT0 from Mazor et al., 2013 and 1JB0 from Jordan et al., 2001) are colored cornflower blue and goldenrod, respectively. Water molecules identified in the higher plant PSI structure (PDB ID: 5L8R from Mazor et al., 2017) are colored yellow green.

Owing to the high resolution, the new structural data allows us to observe more water molecules in both PSI-ACPI and PSII-ACPII. In total, 422 water molecules are found in the PSI-ACPI structure, the majority of which are found mainly in the PSI core part and form two layers, a stromal side layer and a lumenal side layer, whereas very few water molecules are located at the antennae part (**Figure 6A**). At the stromal side, the water molecules form a water cavity close to the binding site of the iron-sulfur cluster, which accommodates three iron-sulfur clusters. In total, 26 water molecules are found in the vicinity (8 Å) of these three iron-sulfur clusters, among which, 17 can be found in cyanobacteria (Jordan et al., 2001; Mazor et al., 2013), and 19 can be observed in higher plants (Mazor et al., 2017; Wang et al., 2021) (**Figure 6B**).

#### 3.3.2 PSII-ACPIIs

There are also a number of water molecules assigned to the density map of the PSII-ACPIIs structure, which are located mainly in the PSII core subunits (**Supplementary Figure S8**). More water molecules are distributed on the lumenal side than those of the stromal side, consistent with the larger area of hydrophilic protein subunits in the lumenal side than that of the stromal side, as well as with the crystal structure of cyanobacterial PSII solved at a higher resolution (Umena et al., 2011; Suga et al., 2015).

### 3.4 Energy transfer pathways

#### 3.4.1 PSI-ACPIs

In the PSI-ACPI structure, we observed the same number of Chls *c* as that in the structure of *R. salina* (8WM6) (Zhang et al., 2024a), whereas two Chls *a* are missing in our structure compared with that in *R. salina* (Zhang et al., 2024a), and four are missing compared with that in *C. placoidea* (7Y7B) (Zhao et al., 2023) (**Figure 2A**; **Supplementary Table S4**). Most of the missing Chls are in the antennae portion of the structure, which has higher mobility than the PSI core. Energy harvested by ACPI-i may be transferred to PsaQ through a pair of Chls *a* (Chl a306/i and Chl a303/Q) at a distance of 17.9 Å in *R. salina* (Zhang et al., 2024a), but this pathway disappeared in our structure due to the loss of Chl a303/Q in the present structure. Another missing Chl a855/A which is found in *R. salina* is also absent in *C. placoidea* (Zhao et al., 2023), and this Chl has a distance of 12.5 Å to Chl a606/c and therefore may receive energy harvested by ACPI-c in *R. salina* only.

**Figure 7:**
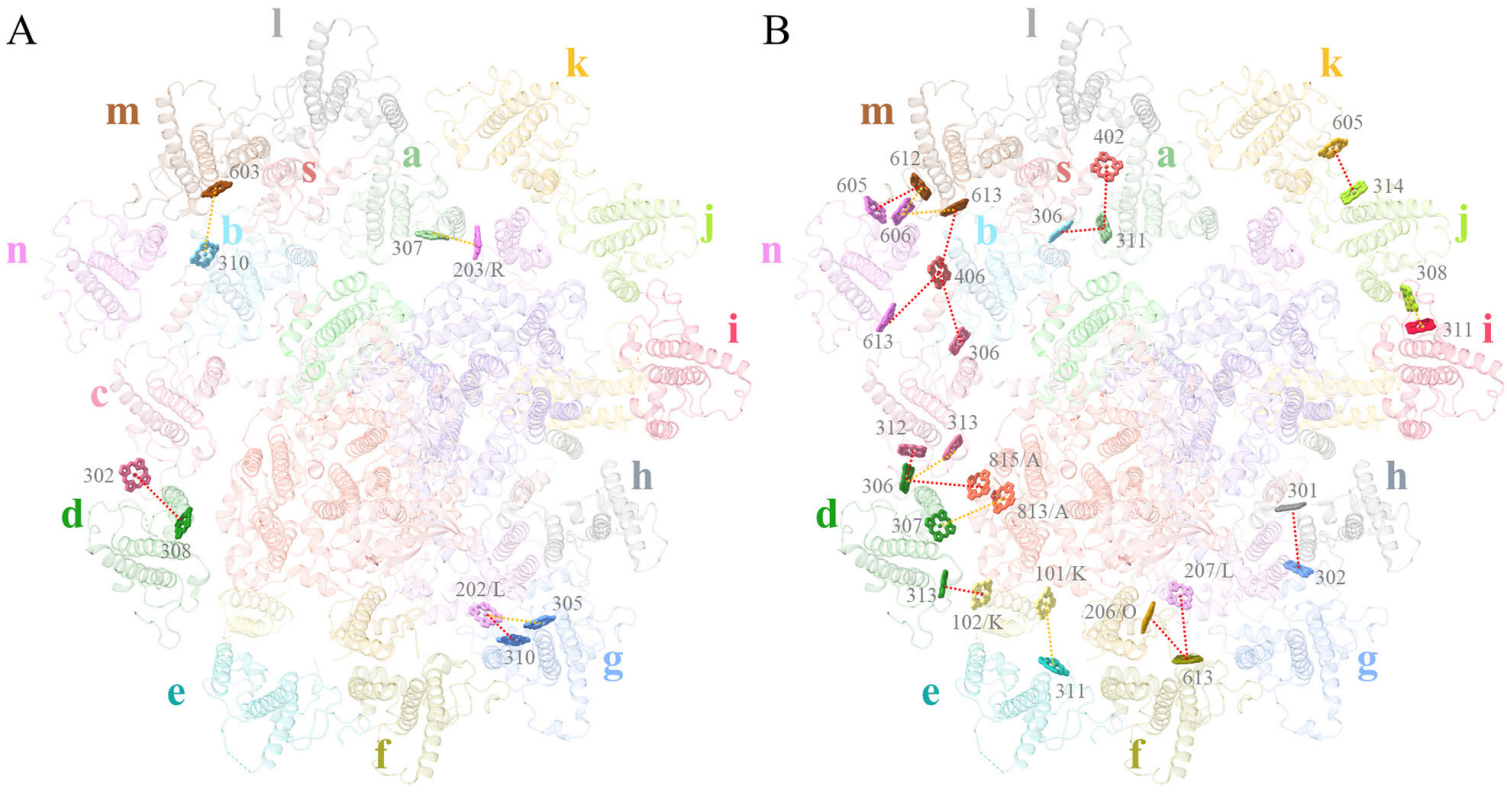
Energy transfer pathways in the *R.* sp. NIES-2332 PSI-ACPI supercomplex. View of Chls involved in the energy transfer pathways on the stromal side **(A)** and luminal side **(B)**. Dashed lines indicate that the FRET rates between Chls are either increased (orange) or decreased (red), compared with those in *R. salina* and *C*. *placoidea*. Chls that are found the same in the three structures are omitted.

For comparing the energy transfer rates in the three cryptophytes, we set the center-to-center distances to 20 Å between neighbouring Chls to calculate them. From antennae subunits to the PSI core, 3 pathways are absent in *R*. sp. NIES-2332, among which, two can be seen in *R. salina* (*a*306_ACPI-d_-*a*815_PsaA_, *a*613_ACPI-n_-*a*406_ACPI-s_), and one is observed in *C. placoidea* (*a*613_ACPI-f_-*a*206_PsaO_) (**Figures 7A, B**; **Supplementary Table S6**). On the other hand, 7 energy transfer pathways are seen in *R*. sp. NIES-2332 but absent in *R. salina* (*a*307_ACPI-a_-*a*203_PsaR_, *a*307_ACPI-d_-*a*813_PsaA_, *a*311_ACPI-e_-*a*101_PsaK_, *a*305_ACPI-g_-*a*202_PsaL_, *a*313_ACPI-d_-*a*102_PsaK_, *a*613_ACPI-f_-*a*207_PsaL_, *a*310_ACPI-g_-*a*202_PsaL_), and 3 out of the 7 are also absent in *C. placoidea* (*a*307_ACPI-d_-*a*813_PsaA_, *a*311_ACPI-e_-*a*101_PsaK_, *a*305_ACPI-g_-*a*202_PsaL_). The other 4 pathways have either increased rate (*a*307_ACPI-a_-*a*203_PsaR_) or decreased rates (*a*313_ACPI-d_-*a*102_PsaK_, *a*613_ACPI-f_-*a*207_PsaL_, *a*310_ACPI-g_-*a*202_PsaL_) in our structure than those in the *C. placoidea* structure (**Figures 7A, B**; **Supplementary Table S6**).

There are also differences in the energy transfer rates among antennae subunits in the three species. This kind of change occurs mostly on the lumenal side, where 3 pathways are absent in *R.* sp. NIES-2332 and *R. salina* but present in *C. placoidea* (*a*302_ACPI-c_-*a*308_ACPI-d_, *a*306_ACPI-c_-*a*406_ACPI-s_, *a*301_ACPI-h_-*a*302_ACPI-g_) (**Figures 7A, B**; **Supplementary Table S6**). Eight pathways are found in *R.* sp. NIES-2332 and *C. placoidea* (*a*311_ACPI-a_-*a*402_ACPI-s_, *a*311_ACPI-a_-*a*306_ACPI-b_, *a*314_ACPI-j_-*a*605_ACPI-k_, *a*612_ACPI-m_-*a*605_ACPI-n_, *a*613_ACPI-m_-*a*406_ACPI-s_, *a*313_ACPI-c_-*a*306_ACPI-d_, *a*612_ACPI-m_-*a*606_ACPI-n_, *a*613_ACPI-m_-*a*606_ACPI-n_) with either increased or decreased rates, but these pathways are absent in R. *salina* (**Figures 7A, B**; **Supplementary Table S6**). One pathway is observed in *R.* sp. NIES-2332 and *R. salina* but absent in C. *placoidea* (*a*311_ACPI-i_-*a*308_ACPI-j_), and finally 2 pathways are observed in the three species with different rates (*a*312_ACPI-c_-*a*306_ACPI-d_, *a*310_ACPI-b_-*a*603_ACPI-m_) (**Figures 7A, B**; **Supplementary Table S6**). Amon these changes, only one pathway occurs in the stromal side (*a*310_ACPI-b_-*a*603_ACPI-m_), and the rest occurs in the lumenal side, suggesting that the antennae subunits in the luminal side are more flexible.

#### 3.4.2 PSII-ACPIIs

**Figure 8:**
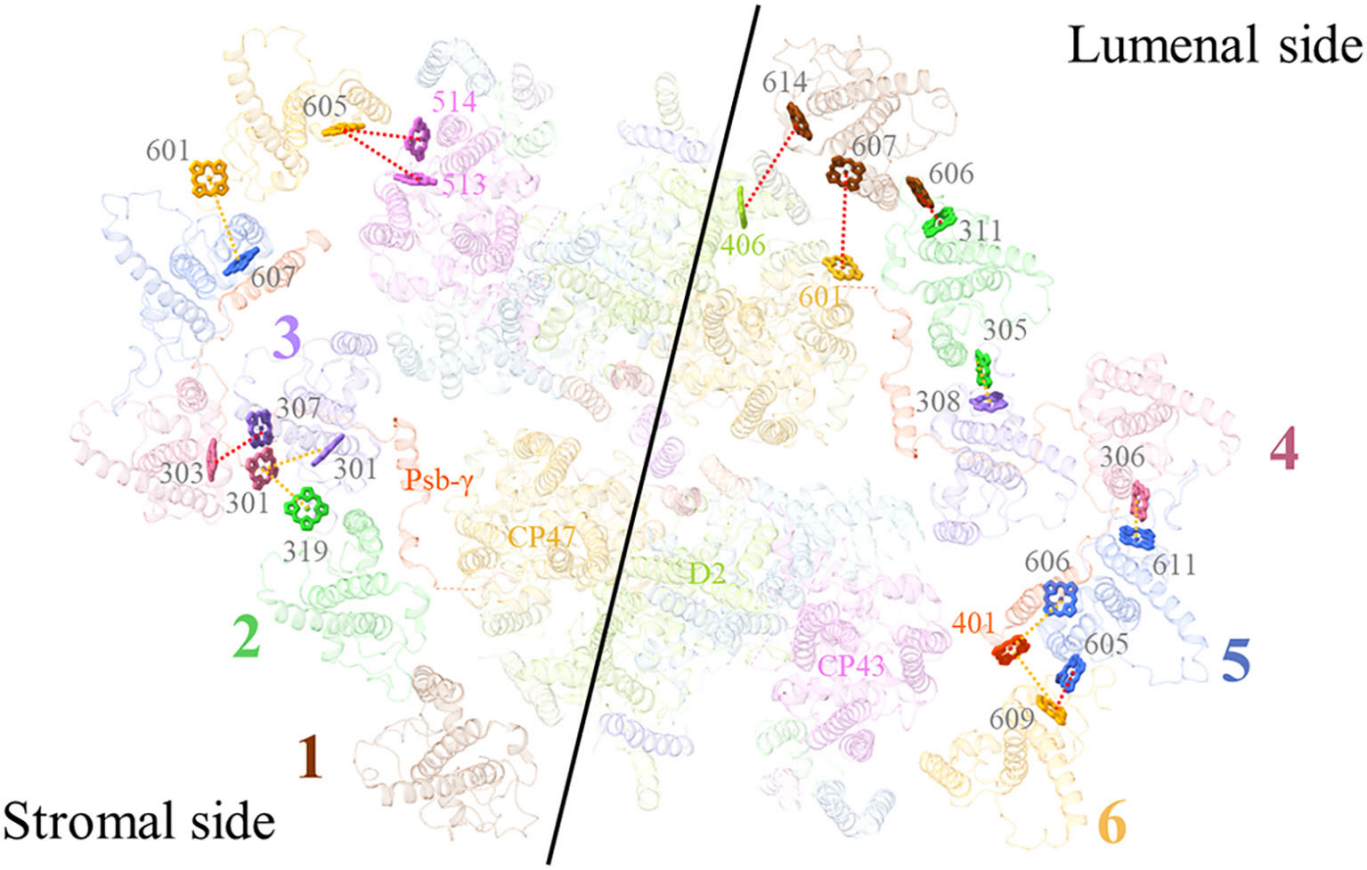
Excitation energy transfer pathways in *R.* sp. NIES-2332 PSII-ACPIIs supercomplex. View of Chls involved in energy transfer pathways from the stromal side and luminal side. Dashlines indicate that the FRET rates between Chls are either increased (orange) or decreased (red) compared with *R. salina* and *C. placoidea*. Chls that are found the same in the three structures are omitted.

There are also differences in the energy transfer pathways and rates in the PSII-ACPII supercomplex among the three species of cryptophytes. From antennae subunits to the PSII core, 2 pathways (*a*607_ACPII-1_-*a*601_CP47_, *a*615_ACPII-1_-*a*404_D2_) are found in *R. salina* but absent in *R.* sp. NIES-2332 and *C. placoidea* (**Figure 8**; **Supplementary Table S7**). Two pathways (*a*606_ACPII-6_-*a*512_CP43_, *a*606_ACPII-6_-*a*513_CP43_) can be observed in *C. placoidea* but absent in *R.* sp. NIEW-2332 and *R. salina*, and 2 pathways are found in *R.* sp. NIES-2332 and *C. placoidea* but absent in *R. salina* (*a*606_ACPII-5_-*a*401_Psb-γ_, *a*609_ACPII-6_-*a*401_Psb-γ_) (**Figure 8**; **Supplementary Table S7**).

Among the antennae subunits, 3 energy transfer pathways are observed in *R.* sp. NIES-2332 but absent in the other two cryptophytes (*a*607_ACPII-5_-*a*601_ACPII-6_, *a*319_ACPII-2_-*a*301_ACPII-4_, *a*301_ACPII-3_-*a*301_ACPII-4_). One pathway is seen in *R.* sp. NIES-2332 and *C. placoidea* with different rates (*a*306_ACPII-4_-*a*611_ACPII-5_), but this pathway is absent in *R. salina* (**Figure 8**; **Supplementary Table S7**). Finally, 4 pathways are observed in all three species but with different rates (*a*307_ACPII-3_-*a*303_ACPII-4_, *a*606_ACPII-1_-*a*311_ACPII-2_, *a*605_ACPII-5_-*a*609_ACPII-6_, *a*305_ACPII-2_-*a*308_ACPII-3_) (**Figure 8**; **Supplementary Table S7**). These differences may either be due to species differences or due to different resolutions of the structures solved, which may give rise to some miss assignments of pigments or positional errors in the structure. In this respect, we would like to point out that the structures we solved here have the highest resolutions, so errors may be smaller in our structure.

## 4 Discussion

The plastid of cryptophytes is considered to originate from a red alga by secondary endosymbiosis (Gentil et al., 2017). Structures of the photosystems from the red linage show that the PSI and PSII core part is largely similar, and the antennae portion also contains some similarity. As the binding module, similar trimers can be observed in the antenna of both PSI and PSII of red algae and diatoms (Xu et al., 2020; Pi et al., 2018; Zhao et al., 2023; Zhang et al., 2024a). For example, in the PSI-ACPI supercomplex of *R. salina* (Zhang et al., 2024a), *C. placoidea* (Zhao et al., 2023) and *R.* sp. NIES-2332 (present structure), four groups of ACPI trimers are observed, among which, ACPI-e/f/g attach to the PSI core at the PsaK, PsaO and PsaL side and have the smallest variations among the three species. In every trimer of ACPIs, the third subunit varies larger, which is relatively flexible than the first and second subunits. Similarly, ACPII are divided into two trimers, ACPII-1/2/3 and ACPII-4/5/6, which are attached to the two sides of the PSII core.

In the PSI-ACPI and PSII-ACPII structures of *R*. sp. NIES-2332, we found that the positions and types of some pigments and lipids are different from those in the structures of PSI-ACPI and PSII-ACPII from *R. salina* and *C. placoidea* solved previously. These differences may partly be due to different resolutions achieved for the three species, but may partly be due to species differences. In any cases, our present structures have higher resolutions, which allowed us to assign the phytol tails of Chls that are previously unable to assign, and differentiate the types of lipids, carotenoids, etc. We found that most of the newly assigned co-factors have interactions with the neighboring subunits, ligands and/or water molecules, suggesting that they contribute to the stability and integrity of the whole structure. The resulted energy transfer pathways have also some differences with the previously solved structures, and the major ones of them are described in the “Energy transfer pathways” in detail.

The iron-sulfur clusters F_X_, F_A_ and F_B_ are surrounded by a water shell in cyanobacteria (Jordan et al., 2001), which is largely conserved in higher plants (Mazor et al., 2017; Wang et al., 2021). We found 26 water molecules in the shell in our structure of PSI-ACPI, among which, 22 can be observed in cyanobacteria and higher plants. Among them, 8 water molecules embedded in the core subunits are the most conserved part, while the water molecules outside of the membrane are less conserved. These may suggest the important roles that water molecules may play in stabilizing the nearby residues/co-factors, thereby stabilizing the complex structure.

There are two PSII-ACPII structures (PDB code: 8XR6 and 8WB4) purified from *C. placoidea* when the cells are at L-growth phase (L-phase) (Mao et al., 2024; Zhang et al., 2024b), and one PSII-ACPII structure (PDB code: 8XLP) purified from *R. salina* when the cells are at S-phase (Si et al., 2024). Both structures of *C. placoidea* (8XR6 and 8WB4) contain the oxygen-evolving complex (OEC) which does not exist in the latter due to the depletion of nitrogen in S-phase. Our PSII-ACPII structure also did not contain OEC, suggesting that the cells we used to purify the PSII-ACPII are in the S-phase. On the other hand, PSI-ACPI purified from cells grown in the S-phase does not contain PsaQ (Zhang et al., 2024a), whereas our PSI-ACPI structure contains PsaQ, suggesting that our cells are in the L-phase. This suggests that the S-phase PSII-ACPII and L-phase PSI-ACPI co-existed in the same cells. This may be caused by the fact that the cells we used to purify PSII-ACPII and PSI-ACPI are grown for around 10 days, which may be in the process of L-phase to S-phase transition. This also suggests that the S-phase PSII-ACPII and L-phase PSI-ACPI can co-exist in the same cells, and that PSII-ACPII may enter into the S-phase from the L-phase before PSI-ACPI.

In conclusion, we solved the structures of PSI-ACPI and PSII-ACPII from *R*. sp. NIES-2332 by cro-EM at higher resolutions, which reveal the existence of some new co-factors as well as some differences in the structures of protein subunits and ligands. Importantly, S-phase PSII-ACPII was found to co-exist with L-phase PSI-ACPI, and PSII-ACPII may enter into the S-phase before PSI-ACPI. These results provide more solid structural basis for elucidating the energy transfer and dissipation processes of both PSII-ACPII and PSI-ACPI in cryptophytes, as well as their assembly processes.

## Data Availability

Cryo-EM maps have been deposited in the Electron Microscopy Data Bank under accession codes of EMD-62717, EMD-62656 and EMD-628469L5V, and the atomic coordinates have been deposited in the Protein Data Bank under accession codes of 9L0K, 9KZ9 and 9L5V, for the PSI-11 ACPIs, PSI-14 ACPIs, and PSII-ACPIIs structures, respectively.
