# Supplementary material for: Structural analysis of PSI-ACPI and PSII-ACPII supercomplexes from a cryptophyte alga Rhodomonas sp. NIES-2332
Source: Front Plant Sci. 2025 Nov 27;16:1716939. doi: 10.3389/fpls.2025.1716939 (PMC12695738; doi:10.3389/fpls.2025.1716939)
Supplement: Supplementary file 1 [file DataSheet1.pdf]

Supplementary materials for

Structural analysis of PSI-ACPI and PSII-ACPII supercomplexes from a cryptophyte alga *Rhodomonas* sp. NIES-2332

Wenyue Zhang, Nozomi Yonehara, Mizuki Ishii, Haowei Jiang, Romain La Rocca, Pi-Cheng Tsai, Hongjie Li, Koji Kato, Fusamichi Akita, Jian-Ren Shen

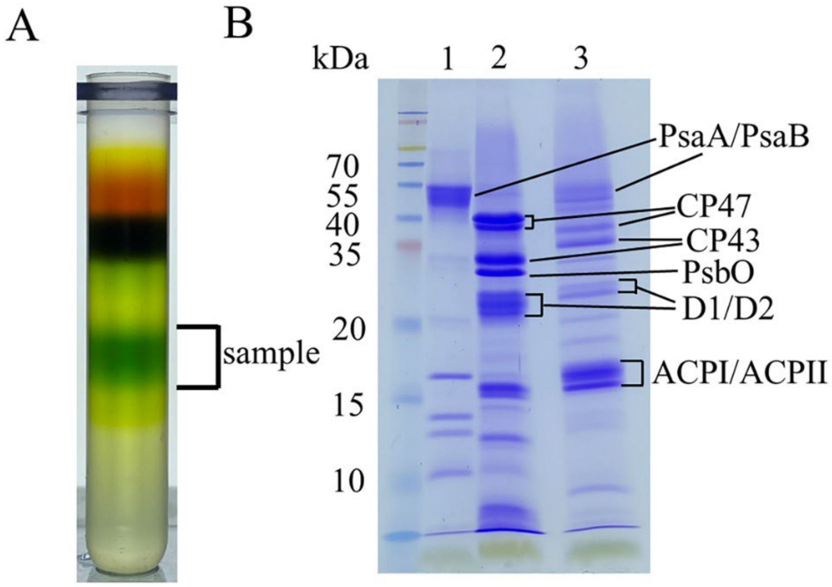

Supplementary Figure S1. Preparation and characterization of the PSI-ACPI and PSII-ACPII supercomplexes from *Rhodomonas* sp. NIES-2332. **A,** Purification of PSI-ACPI and PSII-ACPII by sucrose density gradient centrifugation. The band labeled "sample" is the one that is collected and analyzed subsequently. **B,** SDS-PAGE analysis of the sample collected from the sucrose density gradient centrifugation (PSI-ACPI and PSII-ACPII supercomplexes). Lane 1, PSI from *Thermosynechocossus vulcanus*; lane 2, PSII from *T. vulcanus*; lane 3, PSI-ACPI and PSII-ACPII supercomplexes from *R.* sp. NIES-2332 purified in the present study (the band labeled "sample" in panel A). The major bands of PSI and PSII are labeled according to their molecular weights and previous studies.

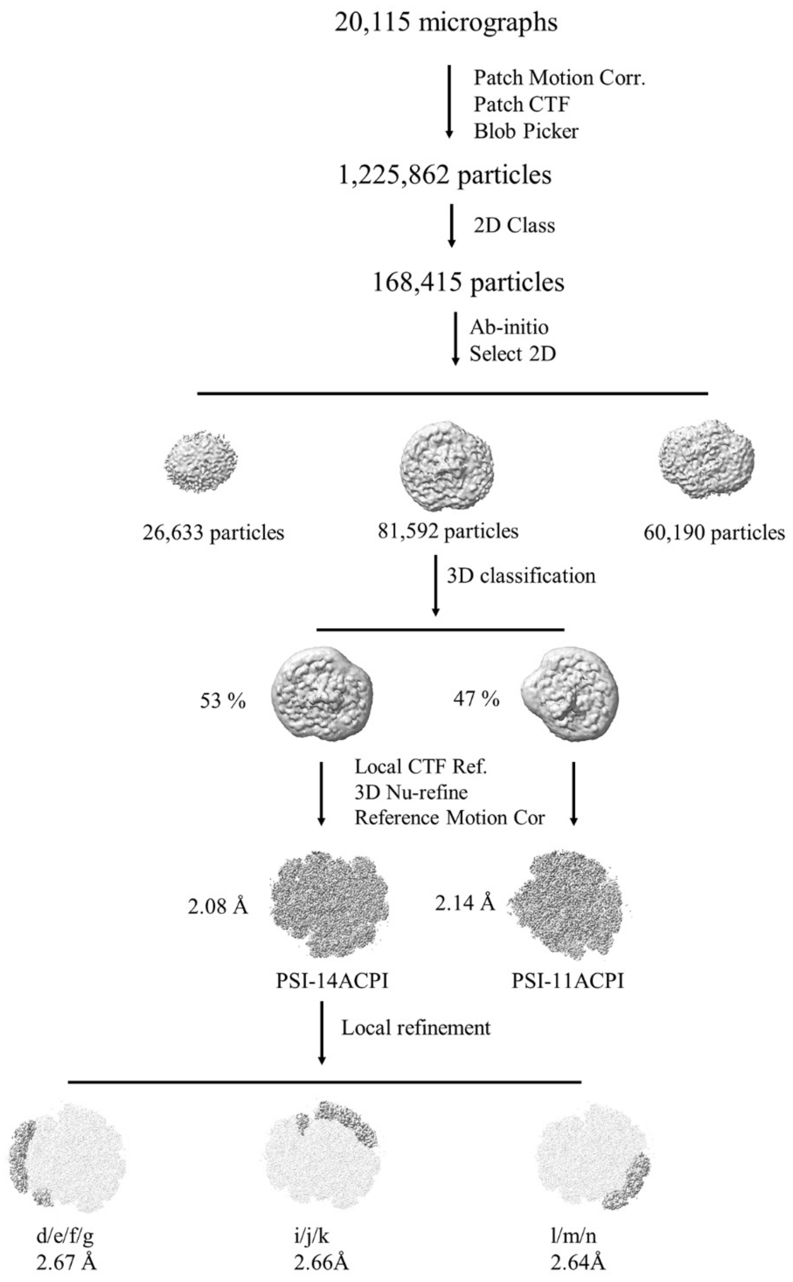

Supplementary Figure S2. Cryo-EM data processing for the PSI-ACPI supercomplex.

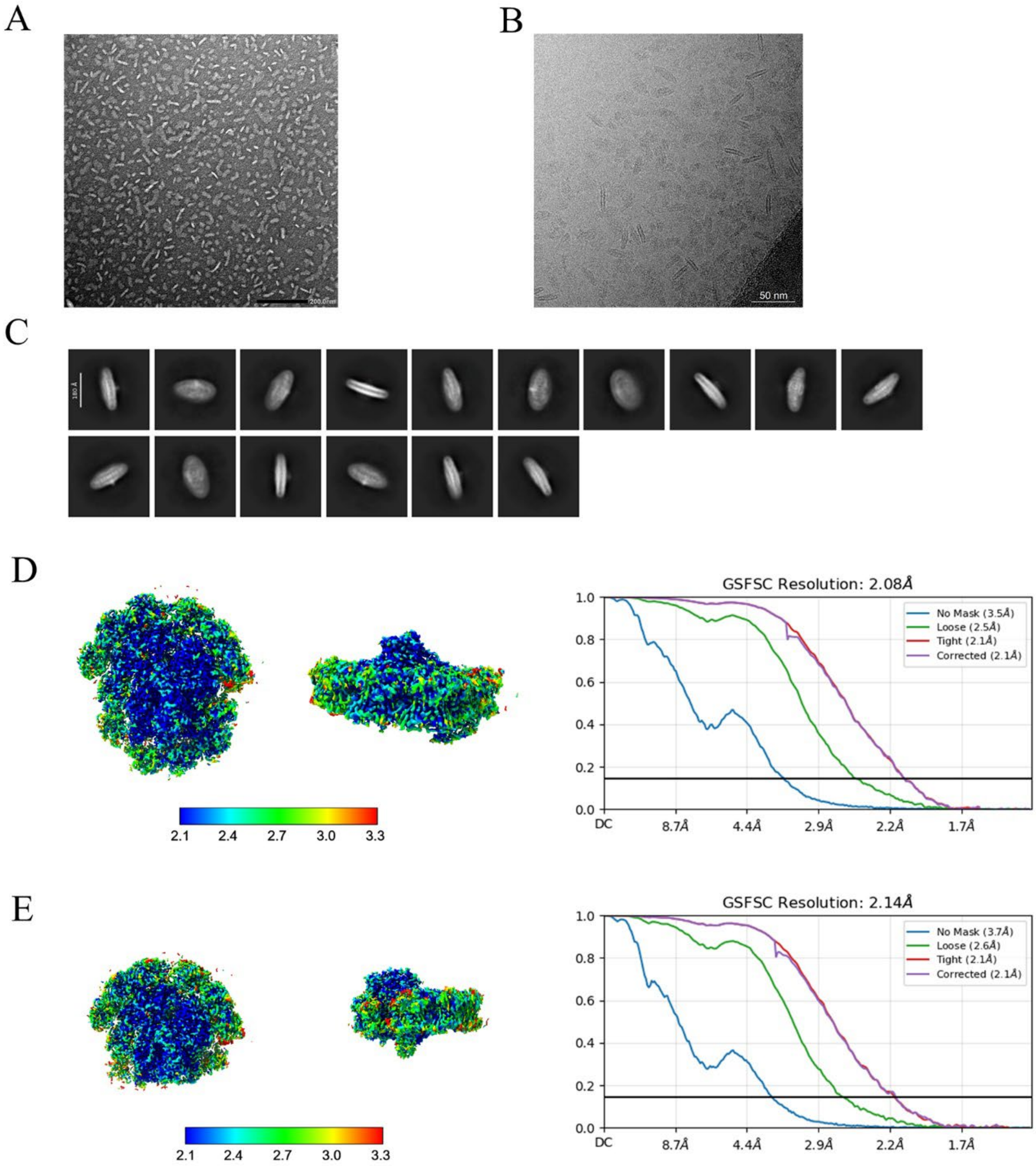

Supplementary Figure S3. Evaluation of the cryo-EM map quality of the PSI-ACPI supercomplex. **A and B,** Representative negative staining micrograph **(A)** and cryo-EM micrograph **(B)** of the *Rhodomonas* sp. NIES-2332 PSI-ACPI supercomplex. **C,** Representative classes of reference-free 2D averages with a particle box size of 600 Å. **D and E,** Local resolution distributions of the cryo-EM map estimated by cryoSPARC and gold-standard Fourier reference (FSC) curves of the final density map with the criterion of 0.143.

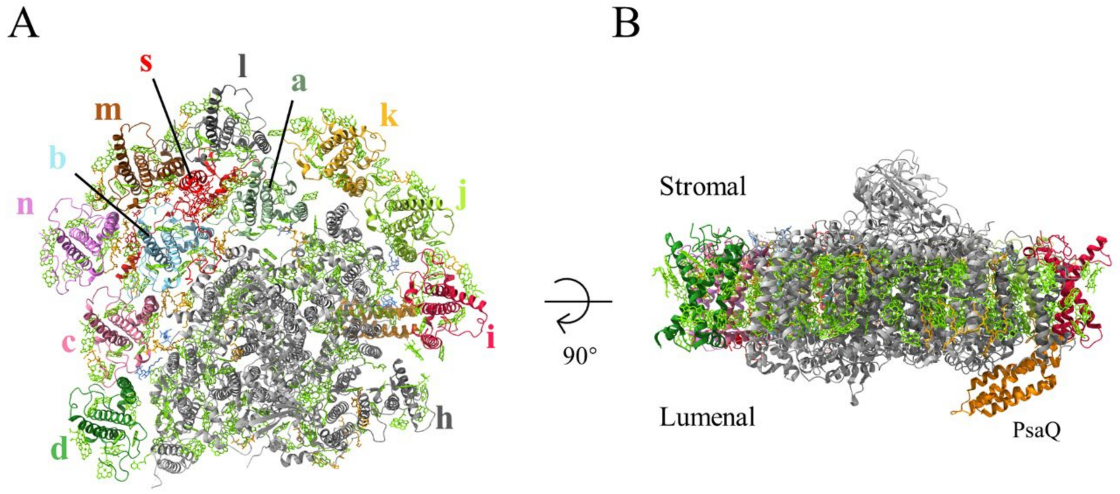

Supplementary Figure S4. Overall structure of the PSI-11 ACPI complex obtained in the present study. **A,** Top view from the stromal side. **B,** Side view. Lawn green: chlorophylls and carotenoids, orange: lipids, cornflower blue: detergents.

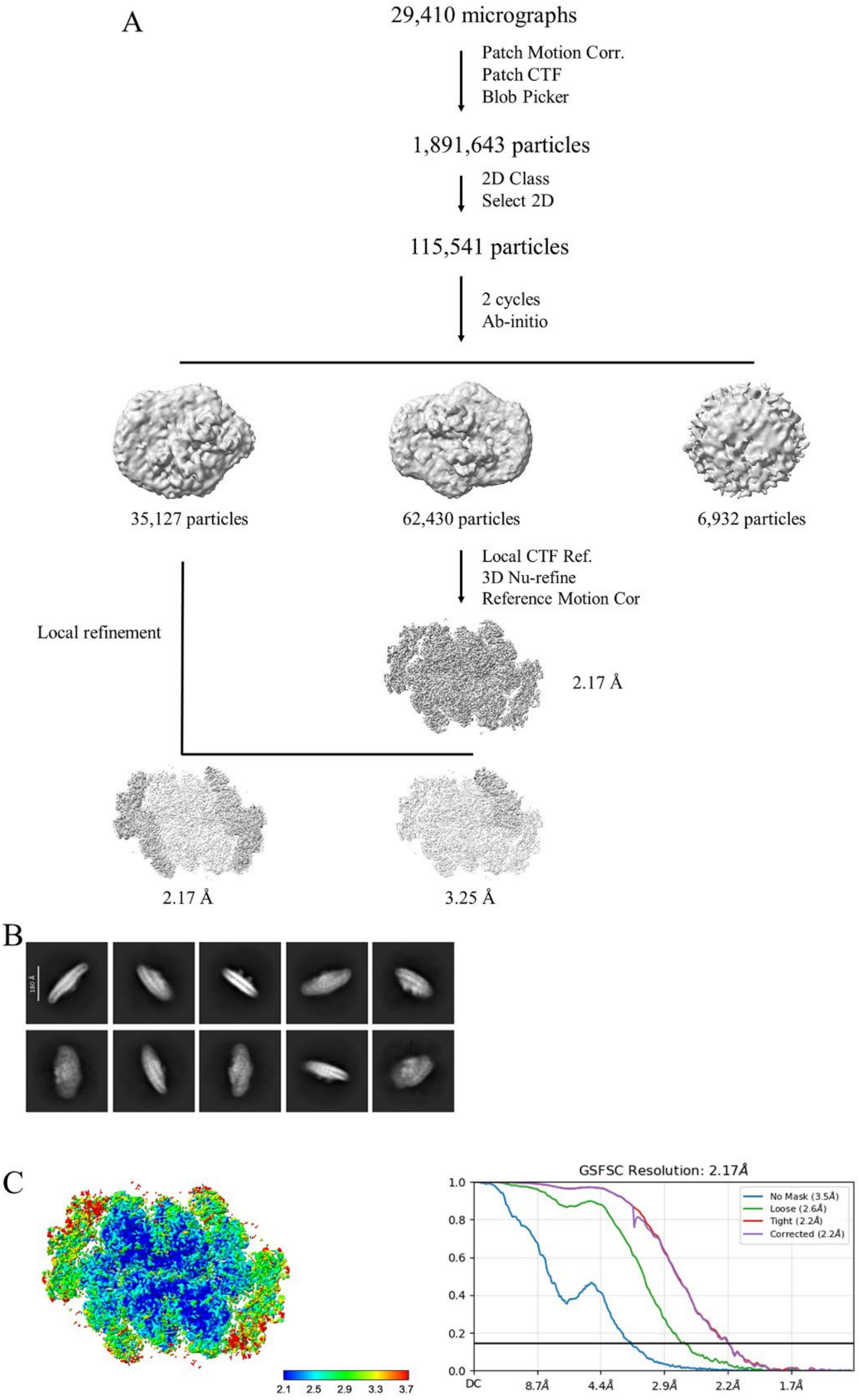

Supplementary Figure S5. Cryo-EM data processing and evaluation of the cryo-EM map quality for the PSII-ACPII supercomplex. **A,** Flow of the cryo-EM data processing. **B,** Representative reference-free 2D class averages with a particle box size of 600 Å. **C,** Local resolution distributions of the cryo-EM map estimated by cryoSPARC and gold-standard Fourier reference (FSC) curves of the final density map with the criterion of 0.143.

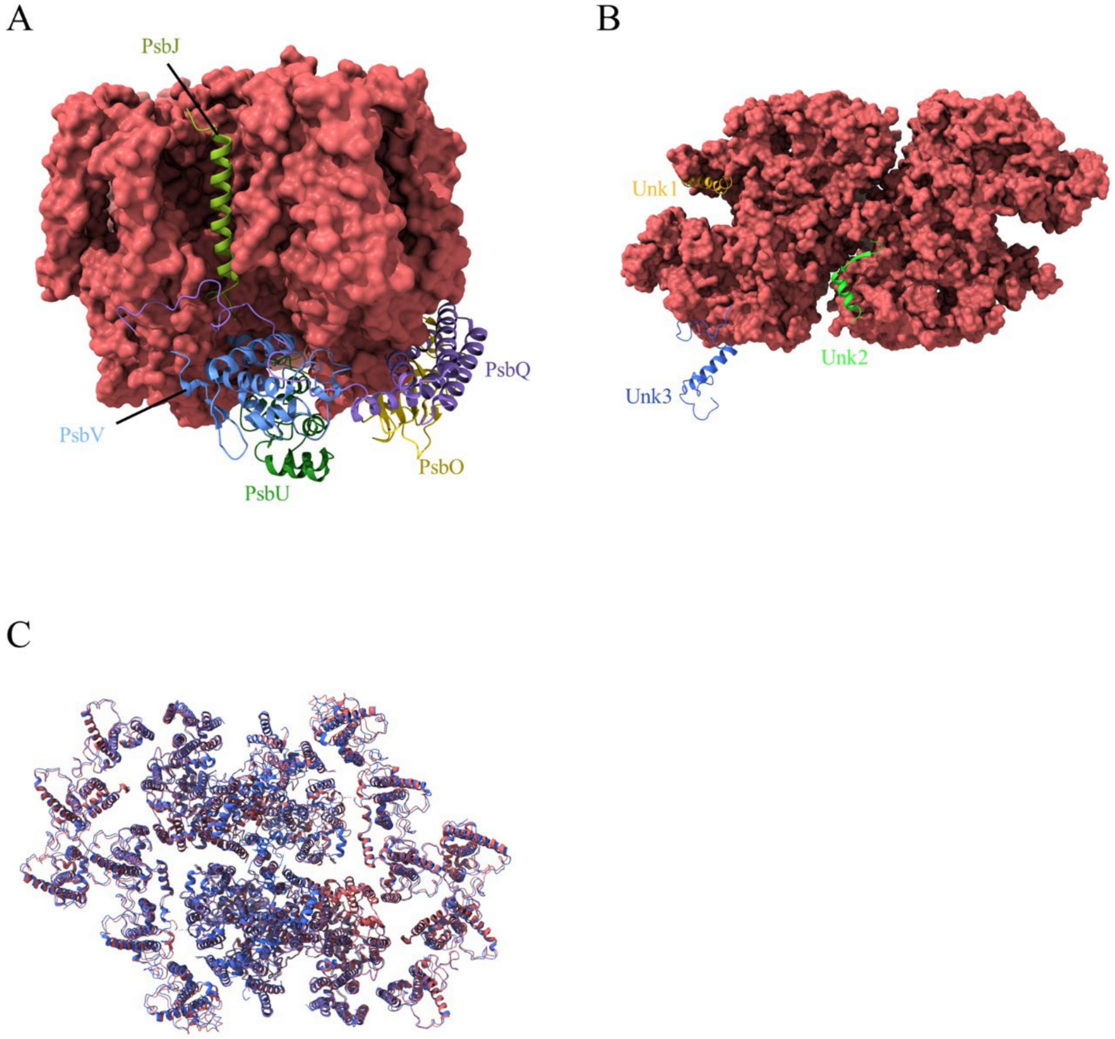

Supplementary Figure S6. A and **B**, PSII core subunits of *R*. sp. NIES-2332 (crimson) compared with *Chroomonas placoidea* (8WB4 from Mao et al., 2024, 8XR6 from Zhang et al., 2024). The Psb J, V, U, O, Q found in *C. placoidea* (Mao et al., 2024; Zhang et al., 2024), and Unk1, 2, 3 subunits found in 8WB4 (Mao et al., 2024) (Psb J, V, U, O are hide) are absent in the structure of *R*. sp. NIES-2332. **C**, Subunit comparison between *R. salina* (8XLP: royal blue) (Si et al., 2024) and *R*. sp. NIES-2332 (crimson) viewed from the stromal side.

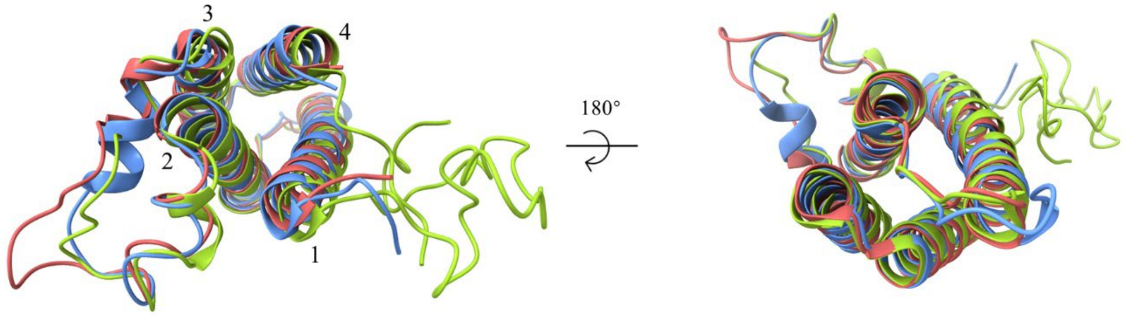

Supplementary Figure S7. Comparison of the position of PsaQ in the structures of three cryptophytes *Rhodmonas* sp. NIES-2332 (indian red), *Rhodomonas salina* (yellow green), and *Chroomonas placoidea* (cornflower blue).

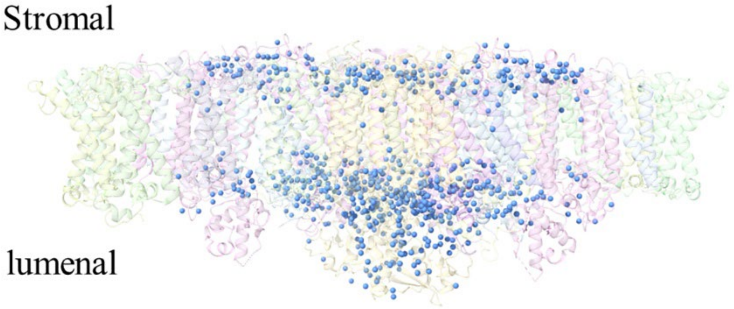

Supplementary Figure S8. Water molecules found in the PSII-ACPII structure of *R*. sp. NIES-2332 (9L5V, cornflower blue). Side view of the structure.

Supplementary Table S1. Cryo-EM data collection and data processing of the PSI-ACPI and PSII-ACPII from *R.* sp. NIES-2332.

	PSI-14ACPIs (9KZ9)	PSI-11ACPIs (9L0K)	PSII-ACPIIs (9L5V)	12 ACPIIs
Data Collection and Processing				
Magnification	165,000			
Voltage (kV)	300			
Electron exposure (e-/Å^2)	50			
Defocus range (μm)	-0.6 ~ -1.8			
Pixel size (Å)	0.727			
Symmetry imposed	C1	C1	C2	C2
Initial particles (No.)	1,225,862	1,225,862	2,844,449	2,844,449
Final particles (No.)	38,563	33,179	28,657	97,557
Map resolution (Å)	2.08	2.14	2.17	2.94
FSC threshold	0.143	0.143	0.143	0.143
Refinement				
Initial model used (PDB code)	8WM6		8XLP	
Map sharpening *B* factor (Å^2)	-20.4	-19.0	-24.0	-50.1
Model composition				
Non-hydrogen atoms	61,391	53,701	40,346	24,565
Protein residues	5195	4544	4107	1937
Ligands	419	354	147	192
B factors (Å^2)				
Protein	55.39	63.27	66.25	128.25
Ligand	56.53	64.78	65.2	117.1
R.m.s. deviations				
Bond length (Å)	0.005	0.004	0.003	0.004
Bond Angels (◦)	1.414	1.072	0.649	0.95
Validation				
MolProbity score	1.34	1.38	1.56	2.05
Clashscore	6.15	6.85	9.36	14.35
Poor rotamers (%)	0.05	0.06	1.23	1.54
Ramachandran plot				
Favored (%)	99.75	99.78	98.53	96.33
Allowed (%)	0.21	0.20	1.47	3.67
Disallowed (%)	0.04	0.02	0	0

Supplementary Table S2. Correspondence of the name of the peripheral antenna of PSI-ACPI in the present paper with those in the PDB files of the other two species of cryptophytes.

R. sp. NIES-2332 (this study) (9KZ9)		*R. salina* (Zhang et al., 2024) (8WM6)		*C. placoidea* (Zhao et al., 2023) (7Y7B)	
Chain ID	Name	Chain ID	Name	Chain ID	Name
/a	ACPI-a	/a	CAC-a	/4	ACPI-4
/b	ACPI-b	/b	CAC-b	/3	ACPI-3
/c	ACPI-c	/c	CAC-c	/2	ACPI-2
/d	ACPI-d	/d	CAC-d	/1	ACPI-1
/e	ACPI-e	/e	CAC-e	/e	ACPI-11
/f	ACPI-f	/f	CAC-f	/d	ACPI-10
/g	ACPI-g	/g	CAC-g	/c	ACPI-9
/h	ACPI-h	/h	CAC-h	/8	ACPI-8
/i	ACPI-i	/i	CAC-i	/7	ACPI-7
/j	ACPI-j	/j	CAC-j	/6	ACPI-6
/k	ACPI-k	/k	CAC-k	/5	ACPI-5
/l	ACPI-l	/l	CAC-l	/b	ACPI-14
/m	ACPI-m	/m	CAC-m	/a	ACPI-13
/n	ACPI-n	/n	CAC-n	/9	ACPI-12
/s	ACPI-s	/s	chain s	/Z	ACPI-S

Supplementary Table S3. Correspondence of the name of the peripheral antenna of PSII-ACPII in the present paper with those in the PDB files of the other two species of cryptophytes.

R. sp. NIES-2332 (this study) (9L5V)	*R. salina* (Si et al., 2024) (8XLP)	*C. placoidea* (Mao et al., 2024) (8WB4)		*C. placoidea* (Zhang et al., 2024) (8RX6)	
Chain ID	Chain ID	Chain ID	Name	Chain ID	Name
1, N	1, N	7, 1	ACPII-1	6, p	ACPII-6
2, O	2, O	8, 2	ACPII-2	5, g	ACPII-5
3, P	3, P	9, 3	ACPII-3	4, 0	ACPII-4
4, Q	4, Q	0, 4	ACPII-4	3, 9	ACPII-3
5, R	5, R	p, 5	ACPII-5	2, 8	ACPII-2
6, S	6, S	P, 6	ACPII-6	1, 7	ACPII-1

Supplementary Table S4. Differences in the pigment-binding sites of PSI-ACPI in the present paper with those in the PDB files of the other two species of cryptophytes.

R. sp. NIES-2332 (this study) (9KZ9)		*R. salina* (Zhang et al., 2024) (8WM6)		*C. placoidea* (Zhao et al., 2023) (7Y7B)	
		PsaA	a855		
		PsaQ	a303		
				ACPI-9	a614
ACPI-a	a306	ACPI-a	a306	ACPI-4	c605
ACPI-b	a313	ACPI-b	a612	ACPI-3	c606
ACPI-d	c312	ACPI-d	c311	ACPI-1	a611
ACPI-e	a305	ACPI-e	a605	ACPI-11	c605
ACPI-j	a307	ACPI-j	a605	ACPI-6	c613
ACPI-k	c612	ACPI-k	c612	ACPI-5	a611
ACPI-k	c613	ACPI-k	c613		
ACPI-l	a307	ACPI-l	a306	ACPI-14	c605
ACPI-n	c612	ACPI-n	c612	ACPI-12	a611
PsaA	α-Car 854	PsaK	α-Car 102	PsaK	Cro 104
PsaB	α-Car 849	PsaB	α-Car 848	PsaB	Alx 843
PsaL	α-Car 201	PsaL	α-Car 201	PsaL	Cro 205
PsaO	Cro 204	PsaO	Cro 204	PsaO	Alx 204
ACPI-a	Cro 316	ACPI-a	Cro 317	ACPI-4	β-Car 616
ACPI-b	Cro 316	ACPI-b	Cro 616	ACPI-3	Alx 617
ACPI-c	Cro 320	ACPI-b	Cro 615	ACPI-3	Alx 615
ACPI-d	Alx 314	ACPI-d	Alx 313	ACPI-1	Mon 615
ACPI-d	Alx 319			ACPI-1	Alx 614
ACPI-d	Alx 316	ACPI-d	Alx 315	ACPI-1	Cro 618
ACPI-e	Alx 313	ACPI-e	Alx 613	ACPI-e	Mon 613
ACPI-g	Cro 324			ACPI-8	Alx 616
ACPI-h	α-Car 308	ACPI-h	α-Car 309	ACPI-8	Cro 609
ACPI-k	Alx 618	ACPI-k	Alx 618	ACPI-5	Cro 618
ACPI-l	Alx 314	ACPI-l	Alx 313	ACPI-14	Mon 613
ACPI-s	α-Car 407	ACPI-s	α-Car 207	ACPI-S	Cro 302

Supplementary Table 5. Differences in the pigment-binding sites of PSII-ACPII in the present paper with those in the PDB files of the other two species of cryptophytes.

R. sp. NIES-2332 (this study) (9L5V)	*R. salina* (Si et al., 2024) (8XLP)	*C. placoidea* (Mao et al., 2024) (8WB4)		*C. placoidea* (Zhang et al., 2024) (8RX6)	
		a102	Unk3		
				a618	CP47
				a304	Psb-γ
				a317	ACPII-5
		a601	ACPII-4	a306	ACPII-3
	c606	c605	ACPII-6	c310	ACPII-1
c605	c605		ACPII-1		
c610	c611	a609	ACPII-1	a310	ACPII-6
c612	c613	a611	ACPII-1	a312	ACPII-6
c304	c606	a605	ACPII-3	c305	ACPII-4
c304	c605	a613	ACPII-4	a315	ACPII-3
c309	c611	a609	ACPII-4	a310	ACPII-3
	Alx 619	Alx 614	ACPII-6	Alx 315	ACPII-1
Alx 610	Alx 617	Mon 615	ACPII-1	Alx 315	ACPII-6
Alx 313	Alx 616	Cro 614	ACPII-4	Alx 317	ACPII-3
Alx 612	Alx 617	Mon 613	ACPII-6	Alx 316	ACPII-1

Supplementary Table S6. FRET rates calculated from the structures of PSI-ACPI of three cryptophytes.

R. sp. NIES-2332 (this study) (9KZ9)		*R. salina* (Zhang et al., 2024) (8WM6)		*C. placoidea* (Zhao et al., 2023) (7Y7B)	
Pigments	FRET rate (ps^{-1})	Pigments	FRET rate (ps^{-1})	Pigments	FRET rate (ps^{-1})
$a306_{\text{ACPI-d}}$-$a815_{\text{PsaA}}$	x	$a305_{\text{ACPI-d}}$-$a815_{\text{PsaA}}$	0.12		
$a613_{\text{ACPI-n}}$-$a406_{\text{ACPI-s}}$	x	$a613_{\text{ACPI-n}}$-$a206_{\text{ACPI-s}}$	0.03	$a612_{\text{ACPI-12}}$-$a301_{\text{ACPI-S}}$	0.00
$a613_{\text{ACPI-f}}$-$a206_{\text{PsaO}}$	x			$a612_{\text{ACPI-10}}$-$a202_{\text{PsaO}}$	0.30
$a307_{\text{ACPI-a}}$-$a203_{\text{PsaR}}$	0.22			$a606_{\text{ACPI-4}}$-$a202_{\text{PsaR}}$	0.08
$a307_{\text{ACPI-d}}$-$a813_{\text{PsaA}}$	1.31			$a606_{\text{ACPI-1}}$-$a812_{\text{PsaA}}$	x
$a311_{\text{ACPI-e}}$-$a101_{\text{PsaK}}$	0.10			$a611_{\text{ACPI-11}}$-$a101_{\text{PsaK}}$	x
$a305_{\text{ACPI-g}}$-$a202_{\text{PsaL}}$	0.55	$a304_{\text{ACPI-g}}$-$a202_{\text{PsaL}}$	x	$a603_{\text{ACPI-9}}$-$a202_{\text{PsaL}}$	x
$a313_{\text{ACPI-d}}$-$a102_{\text{PsaK}}$	0.14			$a612_{\text{ACPI-1}}$-$a102_{\text{PsaK}}$	0.33
$a613_{\text{ACPI-f}}$-$a207_{\text{PsaL}}$	0.02			$a612_{\text{ACPI-10}}$-$a207_{\text{PsaL}}$	0.18
$a310_{\text{ACPI-g}}$-$a202_{\text{PsaL}}$	0.10			$a607_{\text{ACPI-9}}$-$a202_{\text{PsaL}}$	0.22
$a302_{\text{ACPI-c}}$-$a308_{\text{ACPI-d}}$	x			$a601_{\text{ACPI-2}}$-$a607_{\text{ACPI-1}}$	0.34
$a306_{\text{ACPI-c}}$-$a406_{\text{ACPI-s}}$	x			$a605_{\text{ACPI-2}}$-$a301_{\text{ACPI-S}}$	1.19
$a311_{\text{ACPI-a}}$-$a402_{\text{ACPI-s}}$	0.22			$a610_{\text{ACPI-4}}$-$a305_{\text{ACPI-S}}$	0.45
$a311_{\text{ACPI-a}}$-$a306_{\text{ACPI-b}}$	0.12			$a610_{\text{ACPI-4}}$-$a605_{\text{ACPI-3}}$	0.27
$a312_{\text{ACPI-c}}$-$a306_{\text{ACPI-d}}$	0.01	$a611_{\text{ACPI-c}}$-$a305_{\text{ACPI-d}}$	0.08	$a611_{\text{ACPI-2}}$-$a605_{\text{ACPI-1}}$	0.11
$a301_{\text{ACPI-h}}$-$a302_{\text{ACPI-g}}$	x			$a615_{\text{ACPI-8}}$-$a604_{\text{ACPI-8}}$	0.02
$a314_{\text{ACPI-j}}$-$a605_{\text{ACPI-k}}$	0.39			$a611_{\text{ACPI-6}}$-$a613_{\text{ACPI-5}}$	0.87
$a612_{\text{ACPI-m}}$-$a605_{\text{ACPI-n}}$	0.31			$a611_{\text{ACPI-13}}$-$a613_{\text{ACPI-12}}$	0.93
$a613_{\text{ACPI-m}}$-$a406_{\text{ACPI-s}}$	0.27			$a612_{\text{ACPI-13}}$-$a301_{\text{ACPI-S}}$	0.56
$a313_{\text{ACPI-c}}$-$a306_{\text{ACPI-d}}$	1.16			$a612_{\text{ACPI-2}}$-$a605_{\text{ACPI-1}}$	0.50
$a612_{\text{ACPI-m}}$-$a606_{\text{ACPI-n}}$	0.17			$a611_{\text{ACPI-13}}$-$a605_{\text{ACPI-12}}$	0.04
$a613_{\text{ACPI-m}}$-$a606_{\text{ACPI-n}}$	0.86			$a612_{\text{ACPI-13}}$-$a605_{\text{ACPI-12}}$	0.43
$a311_{\text{ACPI-i}}$-$a308_{\text{ACPI-j}}$	0.09	$a311_{\text{ACPI-i}}$-$a606_{\text{ACPI-j}}$	0.03		
$a310_{\text{ACPI-b}}$-$a603_{\text{ACPI-m}}$	0.03	$a609_{\text{ACPI-b}}$-$a603_{\text{ACPI-m}}$	0.01	$a610_{\text{ACPI-3}}$-$a603_{\text{ACPI-13}}$	0.01

Supplementary Table S7. FRET rates calculated from the PSII-ACPII structures of three cryptophytes.

R. sp. NIES-2332 (this study) (9L5V)		*R. salina* (Si et al., 2024) (8XLP)		*C. placoidea* (Mao et al., 2024) (8WB4)	
Pigments	FRET rate (ps^{-1})	Pigments	FRET rate (ps^{-1})	Pigments	FRET rate (ps^{-1})
$a607_{ACPII-1}$-$a601_{CP47}$	x	$a607_{ACPII-1}$-$a601_{CP47}$	1.01		
$a614_{ACPII-1}$-$a406_{D2}$	x	$a615_{ACPII-1}$-$a404_{D2}$	0.87		
$a605_{ACPII-6}$-$a513_{CP43}$	x			$a606_{ACPII-6}$-$a512_{CP43}$	0.12
$a605_{ACPII-6}$-$a514_{CP43}$	x			$a606_{ACPII-6}$-$a513_{CP43}$	0.17
$a606_{ACPII-5}$-$a401_{Psb-\gamma}$	2.29			$a307_{ACPII-5}$-$a302_{CCPII-S}$	1.04
$a609_{ACPII-6}$-$a401_{Psb-\gamma}$	0.56			$a610_{ACPII-6}$-$a302_{CCPII-S}$	0.27
$a607_{ACPII-5}$-$a601_{ACPII-6}$	0.29			$a308_{ACPII-5}$-$a601_{ACPII-6}$	x
$a319_{ACPII-2}$-$a301_{ACPII-4}$	0.03			$a601_{ACPII-3}$-$a608_{ACPII-3}$	x
$a301_{ACPII-3}$-$a301_{ACPII-4}$	0.04			$a602_{ACPII-3}$-$a608_{ACPII-3}$	x
$a307_{ACPII-3}$-$a303_{ACPII-4}$	0.06	$a612_{ACPII-3}$-$a603_{ACPII-4}$	0.14	$a609_{ACPII-3}$-$a603_{ACPII-4}$	0.22
$a606_{ACPII-1}$-$a311_{ACPII-2}$	0.02	$a606_{ACPII-1}$-$a613_{ACPII-2}$	0.05	$a605_{ACPII-1}$-$a611_{ACPII-2}$	0.07
$a605_{ACPII-5}$-$a609_{ACPII-6}$	0.00	$a606_{ACPII-5}$-$a613_{ACPII-6}$	0.06	$a306_{ACPII-5}$-$a610_{ACPII-6}$	0.02
$a305_{ACPII-2}$-$a308_{ACPII-3}$	0.45	$a606_{ACPII-2}$-$a613_{ACPII-3}$	0.11	$a605_{ACPII-2}$-$a610_{ACPII-3}$	0.08
$a306_{ACPII-4}$-$a611_{ACPII-5}$	0.36			$a605_{ACPII-4}$-$a312_{ACPII-5}$	0.05
